# Graded Proteasome Dysfunction in *Caenorhabditis elegans* Activates an Adaptive Response Involving the Conserved *SKN-1* and *ELT-2* Transcription Factors and the Autophagy-Lysosome Pathway

**DOI:** 10.1371/journal.pgen.1005823

**Published:** 2016-02-01

**Authors:** Scott A. Keith, Sarah K. Maddux, Yayu Zhong, Meghna N. Chinchankar, Annabel A. Ferguson, Arjumand Ghazi, Alfred L. Fisher

**Affiliations:** 1 Division of Geriatric Medicine, Department of Medicine, University of Pittsburgh, Pittsburgh, Pennsylvania, United States of America; 2 Division of Geriatrics, Gerontology, and Palliative Medicine, Department of Medicine, The University of Texas Health Science Center at San Antonio (UTHSCSA), San Antonio, Texas, United States of America; 3 Center for Healthy Aging, Barshop Institute for Longevity and Aging Studies, The University of Texas Health Science Center at San Antonio (UTHSCSA), San Antonio, Texas, United States of America; 4 Rangos Research Center, Department of Pediatrics, University of Pittsburgh, Pittsburgh, Pennsylvania, United States of America; 5 San Antonio GRECC, South Texas VA Healthcare System, San Antonio, Texas, United States of America; The University of Texas Health Science Center at Houston, UNITED STATES

## Abstract

The maintenance of cellular proteins in a biologically active and structurally stable state is a vital endeavor involving multiple cellular pathways. One such pathway is the ubiquitin-proteasome system that represents a major route for protein degradation, and reductions in this pathway usually have adverse effects on the health of cells and tissues. Here, we demonstrate that loss-of-function mutants of the *Caenorhabditis elegans* proteasome subunit, RPN-10, exhibit moderate proteasome dysfunction and unexpectedly develop both increased longevity and enhanced resistance to multiple threats to the proteome, including heat, oxidative stress, and the presence of aggregation prone proteins. The *rpn-10* mutant animals survive through the activation of compensatory mechanisms regulated by the conserved SKN-1/Nrf2 and ELT-2/GATA transcription factors that mediate the increased expression of genes encoding proteasome subunits as well as those mediating oxidative- and heat-stress responses. Additionally, we find that the *rpn-10* mutant also shows enhanced activity of the autophagy-lysosome pathway as evidenced by increased expression of the multiple autophagy genes including *atg-16*.*2*, *lgg-1*, and *bec-1*, and also by an increase in GFP::LGG-1 puncta. Consistent with a critical role for this pathway, the enhanced resistance of the *rpn-10* mutant to aggregation prone proteins depends on autophagy genes *atg-13*, *atg-16*.*2*, and *prmt-1*. Furthermore, the *rpn-10* mutant is particularly sensitive to the inhibition of lysosome activity via either RNAi or chemical means. We also find that the *rpn-10* mutant shows a reduction in the numbers of intestinal lysosomes, and that the *elt-2* gene also plays a novel and vital role in controlling the production of functional lysosomes by the intestine. Overall, these experiments suggest that moderate proteasome dysfunction could be leveraged to improve protein homeostasis and organismal health and longevity, and that the *rpn-10* mutant provides a unique platform to explore these possibilities.

## Author Summary

Proteins are complex molecules assembled from individual amino acids that are linked head to tail in a linear chain. Once assembled, the proteins play vital roles in the structure and function of every cell in the body. However, for these proteins to work properly, they must be assembled correctly and resist damage from stresses originating either from inside the body or from the environment. One way that proteins are safeguarded is through the careful removal and destruction of damaged or unwanted proteins via a molecular machine termed the proteasome, which cleaves the protein chain and releases the individual amino acids back into the cell. Usually a loss of proteasome activity leads to a loss of the quality control mechanisms for cellular proteins and can contribute to aging or the development of diseases, such as Alzheimer’s disease. Here we find that when proteasome activity is only partially reduced, several other protein quality control mechanisms are activated, and this actually leads to a net increase in protein quality. This effect could be utilized to help prevent diseases and aspects of aging caused by the accumulation of damaged proteins.

## Introduction

The content and quality of the cellular proteome reflects a balance between the synthesis, folding and refolding, and degradation of individual proteins [1]. Within this framework, the ubiquitin-proteasome system (UPS) plays a key role in maintaining the abundance of cellular proteins via the controlled degradation of selected proteins, and in maintaining the quality of the cellular proteome via the removal of abnormal or damaged proteins [2–4]. The UPS consists of the proteasome, which is a large multi-protein complex made up of two 19S regulatory caps and a 20S catalytic core, and the small 76 amino acid protein ubiquitin. The attachment of ubiquitin to specific lysine residues in a target protein via the sequential actions of ubiquitin-activating enzymes (E1), ubiquitin-conjugating enzymes (E2), and then ubiquitin ligases (E3) serves to target the protein for destruction in the proteasome. The selectivity of the proteasome for ubiquitinated proteins is conferred in part by the 19S subunit that controls access to the 20S catalytic core and has specific subunits that recognize the ubiquitin chains conjugated to proteins [5, 6]. After these subunits bind to the ubiquitin chains, the 19S subunit promotes the deubiquitination and unfolding of the target protein, and then transfers the protein into the 20S core particle for destruction via proteolytic cleavage [7–11]. This proteolytic cleavage proceeds until the protein is cleaved into small peptides of 2–24 amino acids that can diffuse out of the proteasome, and then be degraded by cytoplasmic peptidases [12, 13]. The liberated amino acids can then be either recycled for use in new protein synthesis or be metabolized via intermediary metabolism.

Aging, environmental stress, and a number of disease states are characterized by proteasome dysfunction, when the reserve of proteasome capacity is insufficient to meet cellular needs [14, 15]. The resulting accumulation of mis-folded and damaged proteins could be a direct cause of specific age-related diseases, such as Alzheimer’s disease, and could also be a proximal cause of the aging process [16–19]. Consistent with the potentially grave consequences resulting from the loss of proteostasis, several cellular mechanisms are known to be triggered when the UPS is inhibited, including the activation of the cap’n’collar family transcription factors, such as *skn-1*, Nrf1, and Nrf2, that control the expression of proteasome subunits, the production of proteasome-associated proteins, and the activation of autophagy [20]. The activation of specific cap’n’collar transcription factors is an evolutionarily conserved mechanism to balance the expression level of proteasome subunits to changes in proteasome activity. In *C*. *elegans* the *skn-1*, in *Drosophila* the Nrf2, and in vertebrates the Nrf1 transcription factor promotes the expression of multiple proteasome subunits in response to reductions in proteasome activity [18, 21, 22]. Often in parallel to the increased expression of proteasome subunits, UPS dysfunction leads to the expression of one or more proteasome-associated proteins that bind directly to the 26S proteasome to either increase its catalytic activity, promote proteasome assembly, or relax substrate specificity [20, 23]. For example, in *C*. *elegans* and vertebrates, reductions in proteasome activity lead to the production of the AIP-1 and AIRAP proteins that bind to the proteasome and enhance the removal of damaged proteins from the cell [24, 25]. Interestingly, the expression of both proteasome subunits and *aip-1* is under the control of *skn-1* in *C*. *elegans*, which suggests the existence of a coordinated response to proteasome dysfunction with at least one goal being the rapid compensatory increase in total proteasome capacity [22, 24, 26].

Many of the studies examining the responses to proteasome dysfunction have relied upon the use of chemical proteasome inhibitors or RNAi to produce rapid and marked reductions in proteasome activity. While these treatments produce robust effects, the changes in proteasome activity during aging or the development of age-related disease are, in contrast, likely gradual and only partial. To examine the organismal responses to chronic proteasome dysfunction, we sought to develop a model that would retain some level of proteasome activity and be amenable to genetic and RNAi studies. Here we describe the use of the *C*. *elegans rpn-10* mutant, which lacks the worm ortholog of the Rpn10/PSMD4 proteasome subunit, as a model of chronic proteasome dysfunction [27, 28]. The 19S subunits Rpn10 and Rpn13 act as receptors that recognize the ubiquitin moieties attached to proteins targeted for degradation [29–31]. As in yeast, *rpn-10* is not essential for viability of *C*. *elegans* except when the *rpn-12* subunit is also removed [32]. However, the *rpn-10* mutant does show an accumulation of ubiquitinated proteins and reduced fertility due to feminization of the normally hermaphrodite germline resulting from the failure to degrade the TRA-2 protein via the UPS [27]. We find that this mutant shows evidence of proteasome dysfunction, and as a result of the adaptive response to the reduction in proteasome activity, also unexpectedly becomes long-lived and resistant to threats to the proteome such as heat, oxidative stress, and unstable proteins. To investigate these effects, we use a combination of gene expression studies and transgenic animals to investigate the downstream pathways affected by the *rpn-10* mutation.

## Results

### RPN-10 is expressed broadly and localizes to the cytoplasm and nucleus

**Fig 1:**
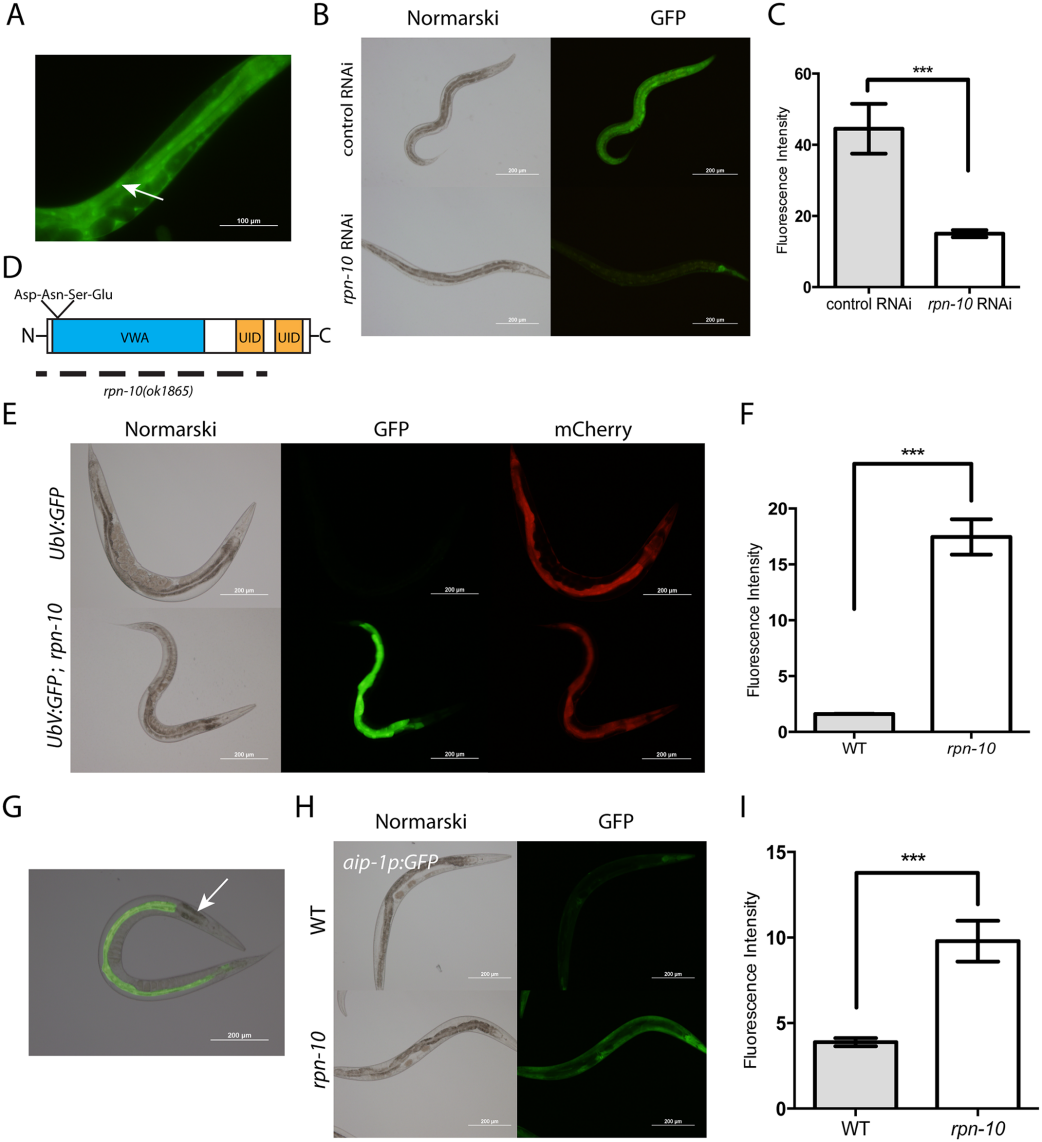
RPN-10 is broadly expressed and contributes to UPS activity. (A) The expression of an RPN-10::GFP fusion protein from a fosmid-based transgene shows broad expression in the cytoplasm and nuclei (arrow) of multiple tissues including the intestine, pharynx, hypodermis, and germline. The GFP signal is produced from the *rpn-10* gene because it can be effectively silenced via treatment of the worms with *rpn-10* RNAi (B). (C) Quantification of GFP expression in digital images captured as in Panel B via the use of ImageJ. *** represents p = 0.001 by *t*-test. (D) Structure of the full-length RPN-10 protein showing the location of the N-terminal Von Willebrand factor A domain (VWA), the location of the highly conserved Asp-Asn-Ser-Glu (DNSE) sequence that is essential for the binding of RPN-10 to the 19S proteasome subunit, and the two C-terminal ubiquitin interacting domains (UID). The dotted line indicates the coding sequence regions deleted in the *rpn-10(ok1865)* allele, and this line extends beyond the N-terminus of the RPN-10 protein to highlight the extension of the deletion into the 5’ UTR of the *rpn-10* gene. (E) The *rpn-10(ok1865)* mutation disrupts UPS function as shown by the selective accumulation of a UbV::GFP fusion protein in the intestine of the mutant but not wild-type animals. In contrast, the *rpn-10* mutation has no effect on the expression of mCherry driven by the same promoter from a separate transgene in the animals. (F) Quantification of GFP expression from digital images captured as in Panel E. *** represents p<0.001 by *t-*test. (G) The accumulated UbV::GFP fusion protein is localized in the intestine except for the three proximal cells (arrow). (H) In addition to accumulating the UbV::GFP fusion protein, the *rpn-10* mutant induces the expression of the *aip-1p*::*GFP* reporter gene in multiple tissues along with the intestine. (I) Quantification of GFP expression from digital images captured as in Panel H. *** represents p<0.001 by *t*-test.

The *C*. *elegans rpn-10* gene encodes the worm ortholog of the Rpn10 proteasome subunit from yeast and the PSMD4 proteasome subunit from vertebrates [28], and worms lacking the *rpn-10* gene are viable but show feminization of the germline [27]. This tissue-specific phenotype observed in the *rpn-10* mutant could suggest that the RPN-10 protein is either expressed in a limited number of tissues in the worm, or that perhaps other proteins or subunits can compensate for the absence of RPN-10. To examine the tissue distribution of RPN-10, we constructed transgenic animals that express an RPN-10::GFP fusion protein from a fosmid clone that has been modified by recombineering to fuse GFP to the C-terminus of RPN-10 [33]. Since the fosmid contains the genomic coding sequence and native promoter, as well as any possible splice variants, the fusion protein is likely both expressed in the proper developmental stages and tissues and trafficked to the correct subcellular locations. The RPN-10::GFP fusion protein was expressed in multiple tissues with the strongest expression seen in the pharynx, intestine, hypodermis, and spermatheca (Fig 1A and 1B). We also observed expression at lower levels in a broader expression pattern, including the excretory cell, body wall muscle, vulva, and somatic gonad, suggesting that the RPN-10 protein might be ubiquitously expressed (S1 Fig). The expression in the spermatheca is consistent with the prior defects noted in sperm development in the *rpn-10* mutant [27]. Additionally, the RPN-10::GFP fusion protein is visible in both the nucleus and cytoplasm (Fig 1A and S1 Fig), suggesting that the proteasome in *C*. *elegans* may play roles in both the cytoplasm and the nucleus as has been noted in other systems [34]. We confirmed that the GFP signal is produced by an RPN-10 fusion protein because treatment of the transgenic worms with *rpn-10* RNAi reduces the expression of GFP in all tissues except for the pharynx (Fig 1B and 1C). The persistent expression of GFP in the pharynx could reflect greater stability of the RPN-10 protein in this tissue compared to others.

### Proteasome dysfunction occurs in the *rpn-10* mutant

Despite the efficient knockdown of RPN-10 expression produced by the *rpn-10* RNAi, we failed to see any gross developmental phenotypes in the treated animals (Fig 1B). Similarly, we noted few developmental phenotypes for the *rpn-10(ok1865)* mutant other than the previously described decline in fertility and a mild increase in time needed to reach adulthood [27]. The *rpn-10(ok1865)* mutation is an 1166 base pair deletion that removes most of the 5’ UTR, the entire first, second, and third exons, and a small portion of the fourth exon of the *rpn-10* gene. This deletion entirely removes the coding sequence for von Willebrand factor type A domain (VWA) and the highly conserved DNSE (Asp-Asn-Ser-Glu) sequence, which together are critical for interactions with other proteasome subunits, and the first of two ubiquitin-interacting motifs (UID) that mediate interactions with ubiquitinated target proteins (Fig 1D). Hence the mutation is expected to be a null or strong loss-of-function [35].

Western blotting has shown that mutants with the *rpn-10(tm1180)* allele, a smaller deletion that removes the third exon and produces a C-terminal truncated protein, accumulate poly-ubiquitinated proteins, which is a phenotype indicating disruption of the UPS [27]. To simultaneously determine whether the *rpn-10(ok1865)* allele impairs UPS activity and to examine the sites of UPS dysfunction, we utilized transgenic animals expressing a UbV::GFP fusion protein under the control of the broadly expressed *sur-5* promoter [36]. This fusion protein is normally rapidly degraded by the UPS, but decreases in UPS activity lead to the accumulation of the fusion protein and result in visible fluorescence [36]. To control for changes in the activity of the *sur-5* promoter or changes in protein translation, the transgenic animals also express a *sur-5p*::*mCherry* transgene. When crossed into the *rpn-10(ok1865)* mutant we observed strong accumulation of the UbV::GFP fusion protein compared to wild-type animals (Fig 1E and 1F), which is consistent with a reduction of UPS activity in this mutant. In contrast to the marked increase in UbV::GFP levels, we saw little to no change in the expression of mCherry, which makes a global effect on transcription or translation unlikely to account for the increase in UbV::GFP (S2 Fig). Additionally, experiments comparing the *rpn-10(ok1865)* mutation to RNAi affecting the 20S proteasome subunits suggests that the impairment of proteasome function shows important distinctions (S3 Fig). Specifically, RNAi knockdown of the 20S catalytic subunit genes *pas-6*, *pbs-6*, or *pbs-7* starting from egg-hatching result in a robust increase in UbV::GFP expression during larval development compared to the *rpn-10* mutant which peaks later. Further the worms treated with *pas-6*, *pbs-6*, or *pbs-7* RNAi either arrest later in development or show harmful effects in the adult animal that are absent in the *rpn-10* mutant [26]. The later accumulation of UbV::GFP and lack of the severe detrimental phenotypes suggests that the *rpn-10* mutant produces a graded reduction in proteasome function compared to the inhibition of the 20S catalytic subunit.

Despite the broad expression pattern of the RPN-10 protein, we observed the accumulation of the UbV::GFP fusion protein selectively in the intestine (Fig 1G). Moreover, within the intestine we observed regional differences with the group of three cells at the proximal end of the intestine showing little accumulation compared to other intestinal cells, despite the expression of mCherry from the *sur-5p*::*mCherry* transgene at this site (Fig 1E and 1G). The reasons accounting for the tissue and cell-specific accumulation of UbV::GFP are not clear, but this could reflect either greater demands for proteasome activity or lower proteasome expression in these areas.

To determine if UPS dysfunction could be occurring in tissues besides the intestine, despite the lack of visible UbV::GFP fusion protein accumulation, we examined the expression of the *aip-1p*::*GFP* reporter gene. The *aip-1* gene encodes an inducible subunit of the proteasome which is selectively activated in the setting of UPS dysfunction produced by a variety of causes [24–26, 37]. We found that the *aip-1p*::*GFP* reporter was activated in a larger range of tissues in the *rpn-10(ok1865)* mutant, including expression in the pharynx, hypodermis, excretory cell, body wall muscle, intestine, and somatic gonad, which suggests that the limited accumulation of the UbV::GFP fusion protein underestimates the degree of UPS dysfunction (Fig 1H and 1I and S4 Fig).

### Loss of *rpn-10* enhances resistance to oxidative stress, thermal stress, and mis-folded proteins

**Fig 2:**
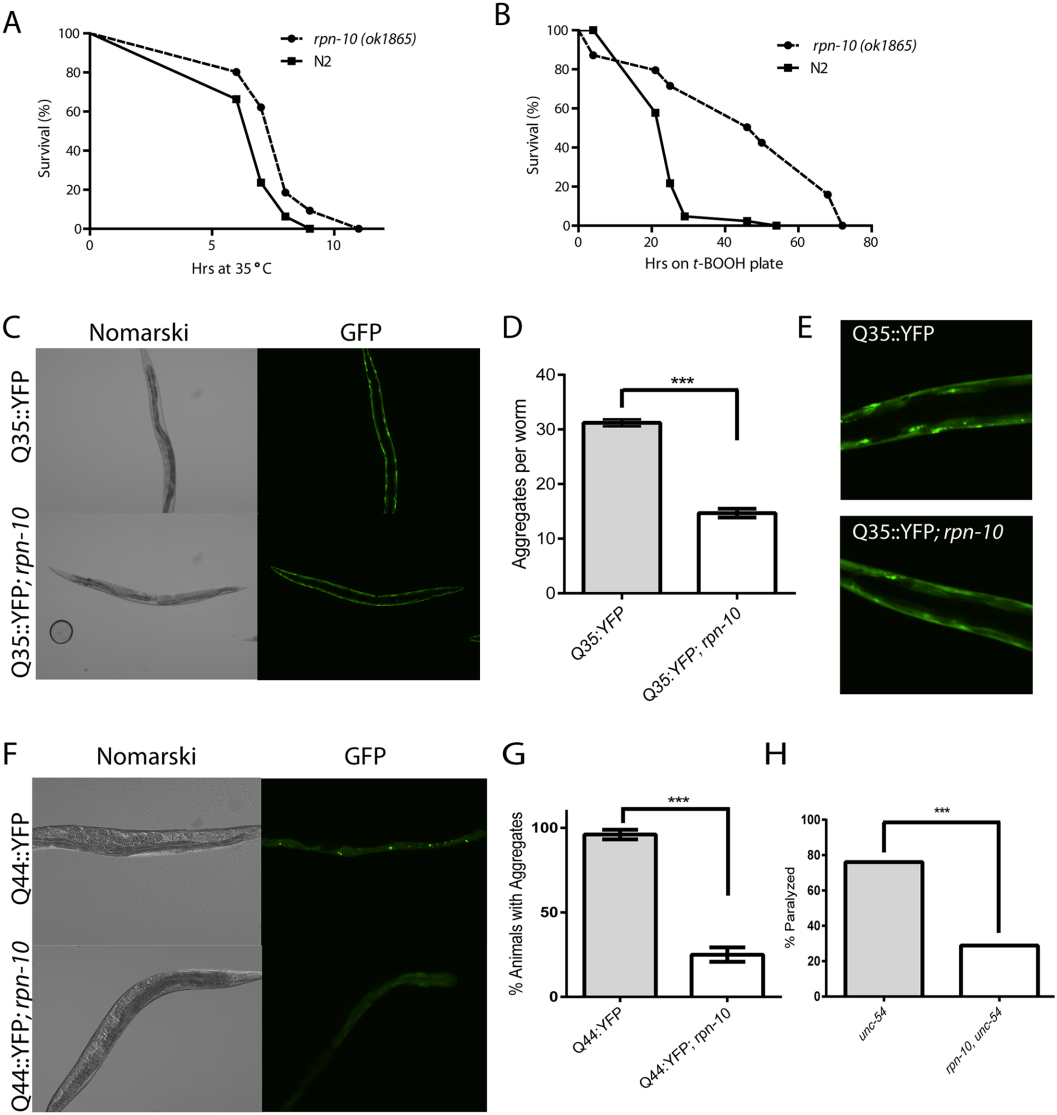
The *rpn-10* mutant shows enhanced resistance to proteostasis threats. Compared to wild-type N2 worms, the adult *rpn-10(ok1865)* mutant shows enhanced resistance to external and internal stresses, including (A) survival during a 35°C heat shock (n = 81 for *rpn-10* and = 80 for N2, p < 0.0001 by log-rank test), (B) survival following exposure to 7 mM *tert*-butyl hydroperoxide (tBHP) (n = 107 for *rpn-10* and = 95 for N2, p < 0.0001 by log-rank test), and the number of aggregates formed when a Q35::YFP fusion protein is expressed in the muscle (C and D) (n = 60 for WT and *rpn-10*, p<0.0001 by *t-*test). The Q35::YFP aggregates seen in the *rpn-10* mutant are also smaller than those seen in the wild-type animals (E). The *rpn-10* mutant also shows a reduction in the percentage of animals showing protein aggregates when a Q44::YFP fusion protein is expressed in the intestine using an integrated transgene (F and G) (n = 100 for WT and *rpn-10*, p < 0.003 by *t*-test). Moreover, the *unc-54(e1157)* mutant is protected from developing paralysis produced by a shift from 16°C (permissive temperature) to 25°C (restrictive temperature) by the *rpn-10* mutation (H) (n = 317 for *unc-54(e1157)* and n = 284 for *rpn-10*, *unc-54*, p < 0.0001 by Fisher’s exact test).

Prior work has demonstrated a role for the UPS system in the response to proteostasis threats including oxidative stress and the expression of mis-folded proteins [38–46]. Given the UPS disruption observed in the *rpn-10(ok1865)* mutant, we tested the ability of this mutant to withstand thermal stress, oxidative stress, and the expression of an aggregation-prone polyglutamine fusion protein. We found that the *rpn-10* mutant showed enhanced survival during a 35°C heat-shock compared to the N2 wild-type control (Fig 2A). This finding is consistent with the increased survival of yeast treated with proteasome inhibitors to survive a subsequent heat shock due to the enhanced expression of heat shock factor proteins [47]. To determine if the *rpn-10* mutant showed a differential resistance to thermal stress compared to oxidative stress, we treated the wild-type animals and the *rpn-10* mutant with *tert*-butyl hydroperoxide (tBHP). While we expected the mutant to be hypersensitive to tBHP, we instead found that the *rpn-10* mutant animals showed a marked increase in survival when exposed to this source of oxidative stress (Fig 2B). Together these findings suggest that the *rpn-10* mutant is better able to resist acute threats to proteostasis compared to a wild-type animal.

To investigate whether the resistance extended to long-term proteostasis threats, we used a transgene to express a Q35::YFP fusion protein in the body wall muscles. Previous work has shown this fusion protein to undergo age-dependent aggregation, which can be modified by changes in proteostasis activity in the cell [41]. Particularly, the inhibition of several proteasome subunits via RNAi is known to increase the aggregation of this protein [41]. In contrast to the effects of acute reductions in proteasome activity, we found that the Q35::YFP fusion protein formed fewer aggregates in the *rpn-10* mutant compared to wild-type animals (Fig 2C and 2D and S5 Fig). Furthermore, the aggregates observed tended to be smaller in the *rpn-10* mutant when observed with fluorescent microscopy (Fig 2E). Since the intestine showed greater evidence of UPS dysfunction than the muscle, we then explored the effects of the *rpn-10* mutant on the aggregation of a Q44::YFP fusion protein that is expressed in the intestine with a transgene [48]. We found that the *rpn-10* mutation served to protect animals from developing polyglutamine aggregates even in the intestine (Fig 2F and 2G and S6 Fig). These last two phenotypes are in contrast to the effects of proteasome inhibition via RNAi, which could reflect either a difference in the degree of UPS disruption in the *rpn-10(ok1865)* mutant, the activation of one or more compensatory pathways, or perhaps a novel role for *rpn-10* outside of the proteasome.

While the reduced aggregation of these polyglutamine-repeat proteins suggests an improvement in proteostasis in the *rpn-10* mutant, a limitation of this experimental approach is that both of the reporters express a non-native protein in the worms. To examine whether the improvement in proteostasis extended to native proteins, we examined the function of the metastable UNC-54 protein that is expressed by the *unc-54(e1157)* mutant [49]. This protein functions normally at the permissive temperature of 16°C whereas at the non-permissive temperature of 25°C, the protein becomes non-functional, presumably due to protein misfolding, and results in the animals becoming paralyzed. We found that the *rpn-10* mutation also prevented the loss of UNC-54 activity when larval animals are acutely shifted to the non-permissive temperature (Fig 2H), perhaps by promoting the stability or folding of the UNC-54 protein. Together these data suggest that the *rpn-10* mutation enhances cellular proteostasis in multiple tissues of the worms.

### Loss of *rpn-10* primes both oxidative and heat-shock responses in *C*. *elegans*

**Fig 3:**
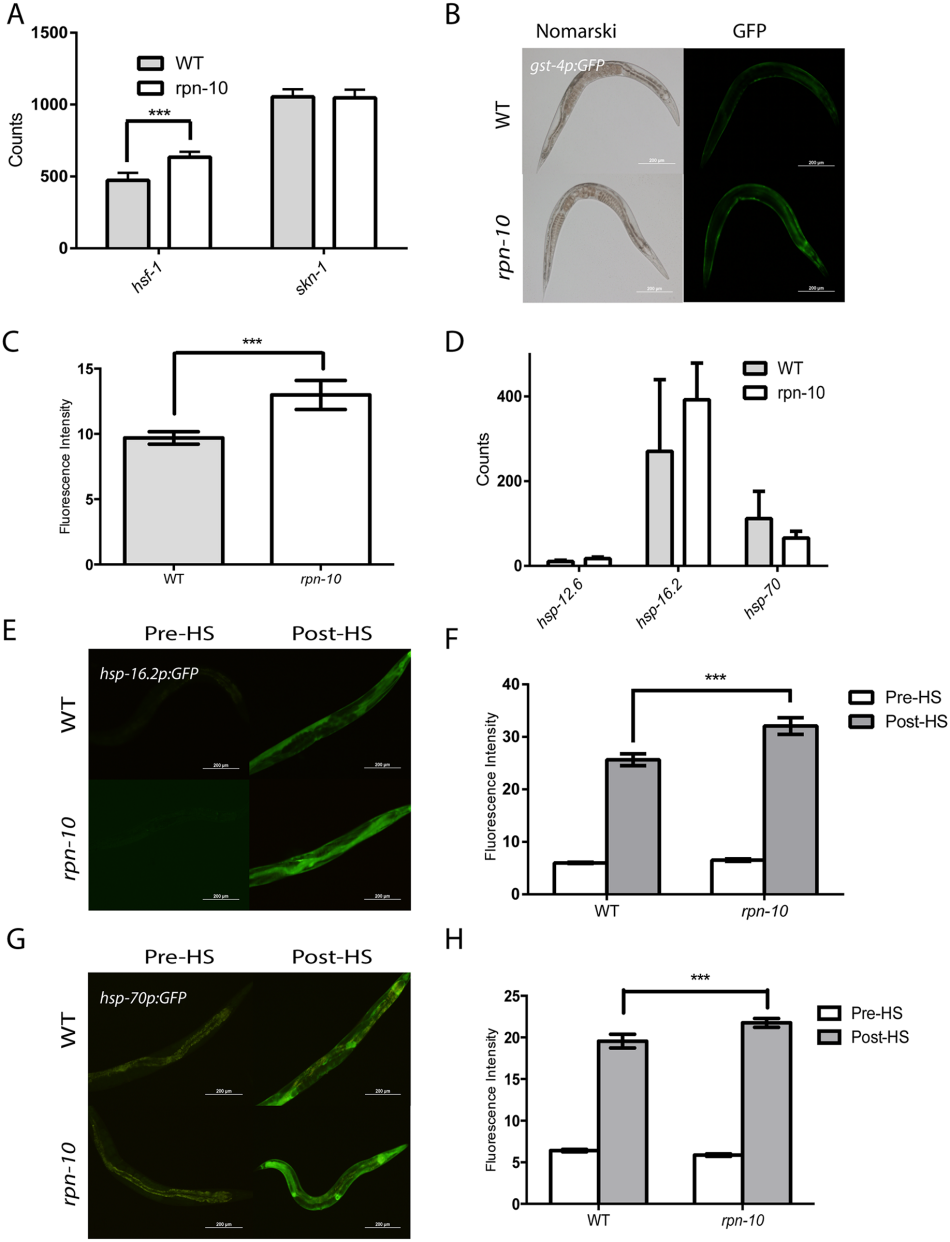
The *rpn-10* mutant shows enhanced expression of oxidative stress and heat shock response genes. (A) The *rpn-10* mutant animals show increased expression of *hsf-1* but not *skn-1* compared to wild-type worms as shown by Nanostring analysis (N = 5 independent samples for N2 and 6 independent samples for *rpn-10*, *** represents p = 0.03). (B and C) The *rpn-10* mutant animals show increased expression of the *gst-4p*::*GFP* reporter compared to wild-type animals, in the absence of exogenous oxidative stress, with increased expression seen mainly in the intestine and hypodermis (n = 15 for WT and *rpn-10*, *** represents p<0.05 by *t*-test). (D) When Nanostring is used to measure the expression of *hsp-12*.*6*, *hsp-16*.*2*, and *hsp-70* in the *rpn-10* mutant and wild-type animals, the *rpn-10* mutants do not show up-regulation of the heat shock response in the absence of external stressors (p>0.2 for all three genes). In contrast, the *rpn-10* mutant animals show increased expression of the *hsp-16*.*2p*::*GFP* (E and F) and of the *hsp-70p*::*GFP* (G and H) reporters during the recovery from a one hour heat shock. In panel F, n = 10 for all genotypes and conditions and p<0.01 for WT and *rpn-10* post-heat shock. In panel H, n = 10 for all genotypes and conditions and p<0.05 for WT and *rpn-10* post-heat shock.

To explore why the *rpn-10(ok1865)* mutant shows increased resistance to oxidative and thermal stress, we examined the expression of the *hsf-1* and *skn-1* transcription factors that control responses to these stresses via the use of Nanostring. We found that the expression of *skn-1* was unchanged whereas the expression of *hsf-1* is increased in the *rpn-10* mutant (Fig 3A) [50, 51]. To examine whether the activity of either transcription factor is changed in the *rpn-10* mutant animals, we tested effects of the mutation on GFP reporters which are known to be triggered by each of the stressors. The *gst-4* gene encodes a member of the glutathione-S-transferase family and was identified as being differentially expressed in worms following exposure to oxidative stress [52]. A GFP reporter controlled by the *gst-4* promoter has similarly been shown to respond to oxidative stress [53]. We found that this reporter is induced in the *rpn-10* mutant even in the absence of oxidative stress, which could suggest that oxidative stress response pathways are activated even in unstressed animals (Fig 3B and 3C). Since we found *hsf-1* to be up-regulated in the *rpn-10* mutant animals, we measured the expression of several heat-shock protein genes controlled by *hsf-1* through the use of Nanostring, and we found a trend towards an increase in the expression of these genes in the *rpn-10* mutant, but the differences failed to reach statistical significance (Fig 3D). We also examined the heat-shock response using GFP reporters for the *hsp-16*.*2* and *hsp-70* genes, which respectively encode an α-crystalline and the inducible isoform of HSP-70 [54, 55]. We found little difference in the expression of either *hsp-16*.*2* (Fig 3E and 3F) or *hsp-70* (Fig 3G and 3H) in the *rpn-10(ok1865)* mutant animals compared to control animals. However, we did see increased expression of both reporters in the *rpn-10* mutant compared to wild-type animals during the recovery from a one hour heat shock (Fig 3E, 3F, 3G and 3H), which is consistent with the *rpn-10* mutant serving to prime the heat-shock response. The cause of this priming is unclear but might reflect the increased expression of *hsf-1* or perhaps the delayed clearance of unfolded proteins.

### The *rpn-10* mutant shows increased longevity

**Fig 4:**
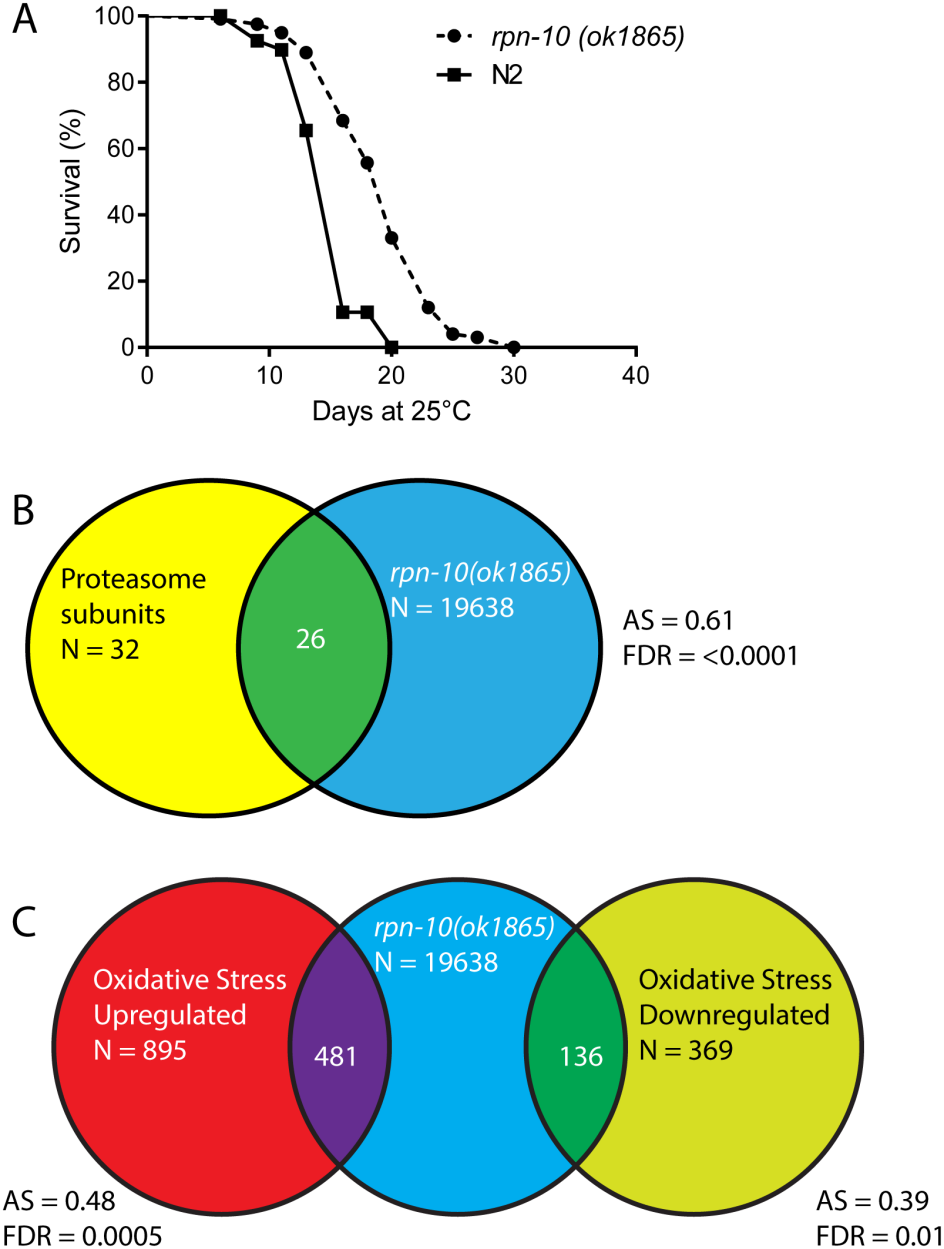
The *rpn-10* mutant shows increased longevity and increased expression of proteasome and oxidative stress response genes compared to wild-type animals. (A) The *rpn-10* mutant has an extended lifespan compared to wild-type animal in lifespan studies conducted at 25°C (mean lifespan 15.0 days for WT (n = 121) and 19.4 days for *rpn-10* (n = 120), p<0.0001 by log-rank test). (B) Shot-gun whole transcriptome sequencing (RNA-seq) was used to characterize and measure the transcriptome of N2 and *rpn-10(ok1865)* mutants. From these experiments, a total of 19,638 mRNA and other RNA transcripts were detected. To test for evidence of a proteasome subunit gene expression signature in the *rpn-10* mutant, Gene Set Association Analysis (GSAA) was used. GSAA calculates a differential expression score for each gene in the entire 19,638 gene RNA-seq dataset, and then uses a running weighted Kolmogorov-Smirnov test to examine association of an entire gene set with each phenotypic class. The strength of the association is measured by the association score (AS) where positive scores indicate association of the gene set with the phenotype, and statistical significance is measured by a false discovery rate (FDR) that is adjusted for multiple testing. From 32 proteasome subunit genes, 26 showed association with the *rpn-10* profile. AS represents the association score with positive values indicating association, and FDR represents the false discovery rate for the association. (C) GSAA provides evidence of an oxidative stress response gene signature in the *rpn-10* mutant. From the 895 genes up-regulated in worms exposed to oxidative stress [60], 481 genes show association with the *rpn-10* profile, and among the 369 down-regulated genes, 136 show an association.

The enhanced proteostasis, oxidative stress responses, and heat shock responses exhibited by the *rpn-10* mutant suggested that these animals could also exhibit an increase in lifespan. However, RNAi studies have demonstrated that the inhibition of most proteasome subunits has a clear detrimental effect on the adult lifespan of *C*. *elegans* [16]. We initially examined the lifespan of the *rpn-10* mutant at 20°C but saw only modest effects, so we then repeated the studies at 25°C based on the observation that the over-expression of the *rpn-6*.*1* subunit only shows a beneficial effect on lifespan at 25°C [56]. At 25°C we observed a consistent increase in the lifespan of the *rpn-10* mutant compared to wild-type animals (Fig 4A and S1 Table) with up to almost a 30% increase in mean lifespan observed. This finding suggests that while reductions in proteasome activity typically have an adverse effect on aging, the net effect of the changes in proteasome activity and the subsequent adaptive responses produced by the *rpn-10* mutation can slow aging and enhance longevity [16, 18, 57].

### RNA-seq analysis demonstrates that proteasome subunits and oxidative stress response genes are over-expressed in *rpn-10* mutants

To identify additional genes controlled by changes in proteasome function, we extracted RNA from three independent samples of *rpn-10(ok1865)* mutants and wild-type animals and used the RNA to identify differentially expressed genes through whole transcriptome sequencing (RNA-seq). From these studies, we identified 19638 distinct RNA transcripts, with 111 genes being differentially up-regulated in the *rpn-10* mutant and 60 genes down-regulated in the mutant (S2 Table). Using the DAVID program to identify themes within the up-regulated and down-regulated genes, we found that proteasome subunits were over-represented among the up-regulated genes (50–70 fold enrichment, p<0.001; S3 Table). This finding was not unexpected as a conserved “bounce-back” response seeks to restore proteasome function in worms or vertebrate cells through the production of additional proteasome complexes [21, 22, 58]. Analysis of the down-regulated genes with DAVID did not identify any over-represented gene classes.

To complement the analysis using the DAVID program, we also tested if specific gene sets showed differential expression in the *rpn-10* mutant through the use of gene set association analysis (GSAA) [59]. With GSAA we found that 26/32 proteasome subunits showed differential expression in the *rpn-10* mutant, and these increases were readily apparent when the expression of individual genes in the RNA-seq data set where examined (Fig 4B and S4 Table). Additionally, our experiments identified enrichment, in the *rpn-10* mutant, of genes previously found to be differentially expressed in worms exposed to oxidative stress produced by hyperbaric oxygen treatment (Fig 4C) [60]. This finding is consistent with the elevated expression of the *gst-4*::*GFP* reporter we observed and suggests that the activation of oxidative stress responses extends to a larger number of genes (Fig 3A). To better visualize the effects of the *rpn-10* mutation on the oxidative stress and heat-shock responses, we examined the expression changes for individual genes involved in each response within the RNA-seq results, and we found increased expression of multiple gene classes within each group (S4 Table). For example within the heat-shock proteins, we again saw the increased expression of *hsf-1* and multiple *hsp-16* proteins while *hsp-1* and most *hsp-12* genes showed little change in expression in the *rpn-10* mutant (S4 Table).

Manual inspection of the differentially expressed genes also revealed possible insights into the response of the mutant worms to the reduction in proteasome activity. The up-regulation of proteasome subunits was clearly present with *pas-3*, *pas-6*, *pas-7*, *pbs-3*, *pbs-6*, *rpt-4*, *rpn-8*, *rpn-9*, and *dss-1*, which is the *C*. *elegans* ortholog of the yeast proteasome regulatory cap protein SEM1, all being overexpressed [61]. We also found that the worm ortholog of NEDD8, *ned-8*, was up-regulated in the *rpn-10* mutant. NEDD8 is a small protein with a highly similar amino acid sequence to ubiquitin (~60% sequence identity) [62, 63]. However, NEDD8 differs functionally from ubiquitin in that NEDD8 is covalently attached selectively to cullin proteins where it promotes the formation of the E2–E3 ligase complex, which then catalyzes the ubiquitination of proteins [64, 65]. However, under conditions of proteasome dysfunction when free ubiquitin levels are low due to the accumulation of ubiquitinated proteins, NEDD8 can also be activated by the same pathways that act to attach ubiquitin to target proteins [66, 67]. The role of NEDD8 in this setting is unclear as this could simply reflect NEDD8 being aberrantly utilized by the ubiquitin activating enzymes due to the low levels of ubiquitin. Alternatively, the use of NEDD8 may constitute part of a response pathway to the reduced cellular proteasome activity [67]. Another notable gene found to be up-regulated in the *rpn-10* mutant was *atg-16*.*2*, which is one of two worm orthologs of ATG16L1, and participates in autophagy via the recruitment of an ATG5-ATG12 complex to the nascent autophagosome [68, 69]. Autophagy and the UPS are known to represent parallel pathways by which protein degradation can occur in the cell, so the responsiveness of *atg-16*.*2* to changes in proteasome function could represent a point of cross-talk between the pathways. Also, among the up-regulated genes is *prmt-1/epg-11* which encodes an arginine methyltransferase that acts to methylate specific cargo receptor proteins and is essential for their clearance of protein aggregates by autophagy during development [70]. We also saw further evidence of an increase in the expression of autophagy genes when the expression of individual genes was queried using the RNA-seq data with 14 out of 21 genes selected showing an increase (S4 Table). Among the down-regulated genes, we identified *cpi-1*, which encodes one of the worm cystatin genes [71]. An important role for cystatins is the inhibition of cathepsins, which are lysosomal proteases that degrade proteins brought to the lysosome via endocytic or autophagic transport pathways [71, 72]. Hence, the down-regulation of *cpi-1* may suggest changes in lysosomal activity that might facilitate the ultimate degradation of proteins that are engulfed by macro or selective-autophagy.

### The *skn-1* transcription factor is required for *rpn-10* mutant survival

**Fig 5:**
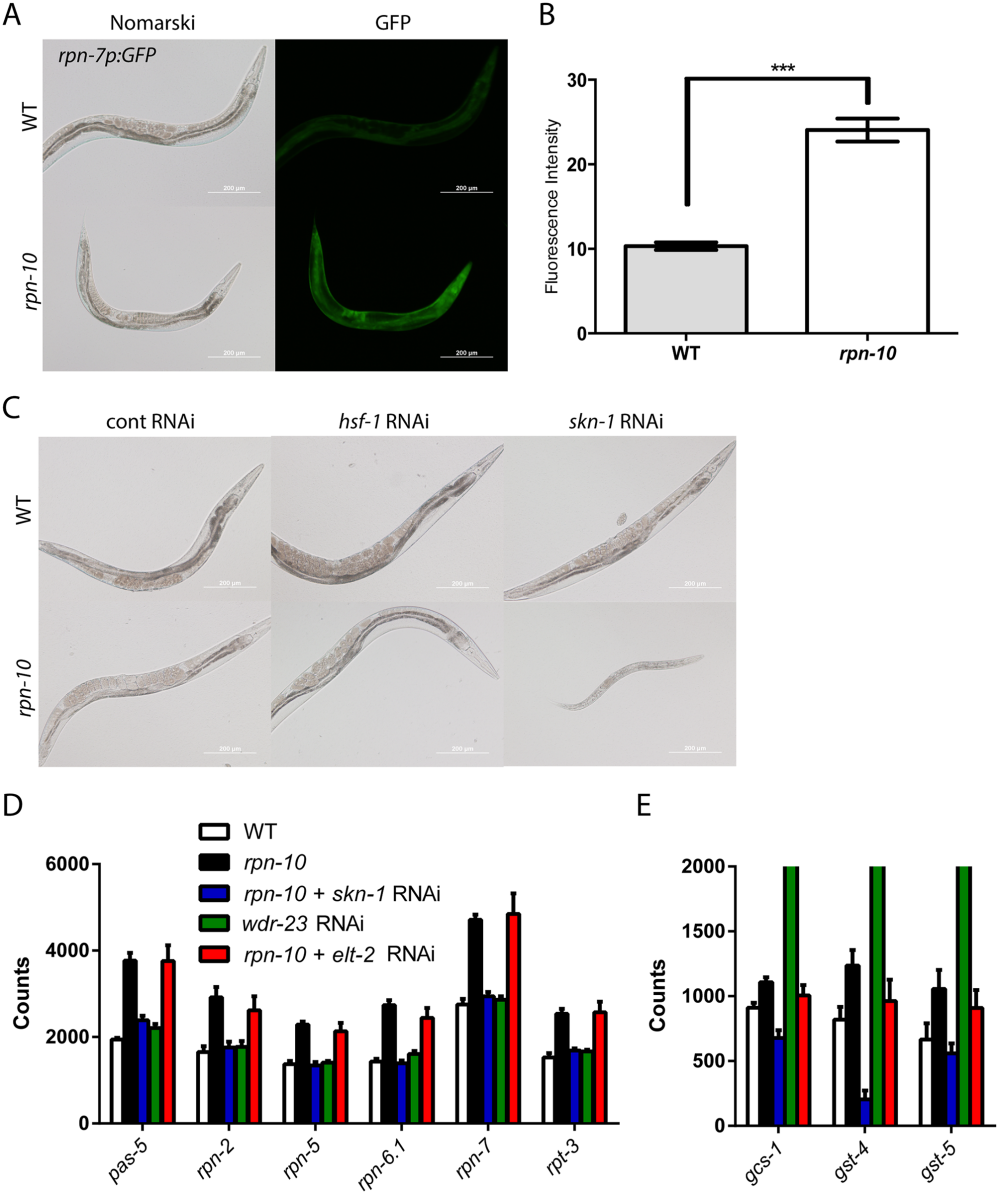
*skn-1* is required for the viability of the *rpn-10* mutant and controls the expression of proteasome subunits. (A) The *rpn-10* mutant shows activation of an *rpn-7p*::*GFP* reporter in multiple tissues including the pharynx, intestine, and hypodermis that is confirmed by quantifying the GFP fluorescence in the images (B) (n = 11 for WT and *rpn-10*, p <0.001 by *t-*test.) (C) Treating the *rpn-10* mutant with *skn-1*, but not *hsf-1* RNAi results in a developmental arrest phenotype. (D) Analysis of gene expression via Nanostring shows an increase in proteasome subunits in the *rpn-10* mutant compared to wild-type animals. The increase in proteasome subunits depends on *skn-1* but not *elt-2*, and the activation of *skn-1* with *wdr-23* RNAi fails to activate subunit expression. (E) In parallel Nanostring studies, the *rpn-10* mutant also shows a small *skn-1* dependent, but not *elt-2* dependent, increase in the expression of the oxidative stress response genes, *gcs-1*, *gst-4*, and *gst-5*. In contrast, *wdr-23* RNAi produces a marked increase in the expression of these genes (mean expression level of *gcs-1–*4,326, *gst-4–*24,714, and *gst-5–*4,982). For details of statistical testing for Panel D and Panel E see S5 Table.

The RNA-seq experiments suggested that both proteasome subunit and oxidative stress response genes are up-regulated in the *rpn-10* mutant. Consistently, we found increased expression of the *gst-4p*::*GFP* oxidative stress reporter in the *rpn-10* mutant (Fig 3B and 3C). To examine the regulation of proteasome subunit expression, we used an *rpn-7p*::*GFP* reporter that expresses GFP under the control of the *rpn-7* promoter. The *rpn-7* gene encodes the worm ortholog of the human PSMD6 protein, which is a subunit of the 19S regulatory cap [28]. In wild-type worms, the *rpn-7p*::*GFP* reporter was expressed in multiple tissues including the intestine, pharynx, and hypodermis (Fig 5A), and the level of GFP expression in these tissues was globally increased in the *rpn-10* mutant (Fig 5A and 5B). Hence despite the focal accumulation of UbV::GFP in the *rpn-10* mutant, multiple tissues in the animal appear to sense changes in proteasome activity and up-regulate the expression of other subunits.

In *C*. *elegans*, the control of oxidative stress responses and the up-regulation of proteasome expression following acute reductions in proteasome activity are both coordinated by the cap’n’collar transcription factor *skn-1*, which is the ortholog of the Nrf1 and Nrf2 transcription factors from vertebrates [22, 50, 73]. We have previously shown that an additional aspect of the response to proteasome dysfunction is the induction of the *aip-1*/AIRAP gene, which encodes an inducible proteasome subunit that enhances proteasome activity and relaxes substrate specificity when bound to the 19S cap, and that this induction requires both *skn-1* and *hsf-1* [24–26]. Hence we sought to determine if *skn-1* and/or *hsf-1* is required for the activation of proteasome subunit expression in the *rpn-10* mutant. However, when we treated the *rpn-10* mutant with *hsf-1* and *skn-1* RNAi, we unexpectedly found that inhibition of *skn-1* resulted in animals that were small, sickly, and developmentally arrested prior to adulthood (Fig 5C). The effect of the *skn-1* RNAi was particularly acute for L3 and L4 larval stage animals because *rpn-10* mutant animals treated with *skn-1* RNAi from egg hatching appeared similar to the control RNAi treated animals on the second day of treatment but then exhibited the detrimental effects on the third day of treatment (S7 Fig). This could reflect the time needed for full effect of the *skn-1* RNAi or the presence of a critical developmental period when *skn-1* activity is essential. In contrast, treatment with *hsf-1* RNAi had no effect on worm development (Fig 5C). This finding demonstrates that while *skn-1* is usually not essential for larval development, *skn-1* is essential for development in the *rpn-10* mutant.

To determine if the essential role played by *skn-1* could be mediated via the control of proteasome subunit expression, we treated wild-type N2 and *rpn-10* mutant animals with control and *skn-1* RNAi, and then measured the expression of several proteasome subunit genes using Nanostring analysis [74]. For these studies, we prepared RNA from the RNAi treated animals at 48 hours after synchronization, which is a timepoint prior to the appearance of any visual phenotypes due to *skn-1* RNAi (S7 Fig). We found that all of the proteasome subunits, for which we probed, were up-regulated in the *rpn-10* mutant compared to N2, and that treatment with *skn-1* RNAi largely prevented this up-regulation (Fig 5D and S5 Table). To determine if SKN-1 activation was sufficient for the increased expression of proteasome subunits, we also treated N2 with *wdr-23* RNAi. The *wdr-23* gene encodes a WD40 repeat protein that binds to *skn-1* and inhibits its transcriptional activity [75]. We found that *wdr-23* RNAi potently increased the expression of oxidative stress response genes (Fig 5E and S5 Table), but had little effect on the expression of proteasome subunits. Conversely, the *rpn-10* mutant showed a greater increase in the expression of proteasome subunits, with a more moderate effect on the activation of oxidative stress response genes (Fig 5D and 5E). Together, these findings suggest that *skn-1* is necessary but not sufficient for the activation of proteasome subunit expression, and that *skn-1* is capable of mounting distinct responses to oxidative stress and proteasome dysfunction.

### *elt-2* is required to survive chronic proteasome dysfunction

**Fig 6:**
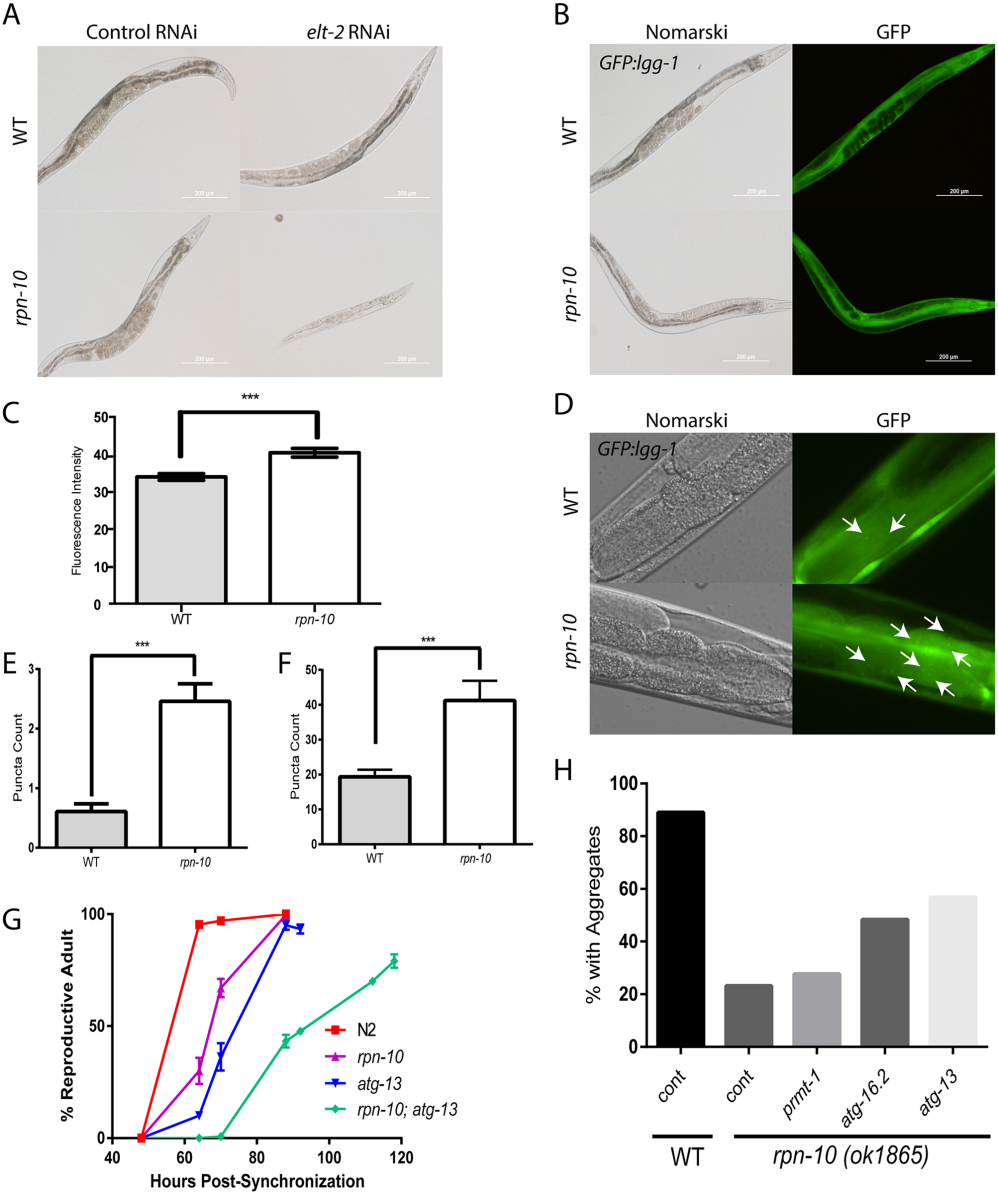
*elt-2* is required for the viability of the *rpn-10* mutant. (A) Treatment of the *rpn-10* mutant, but not wild-type animals with *elt-2* RNAi results in a developmental arrest phenotype. (B) The *rpn-10* mutant shows elevated expression of GFP::LGG-1 as shown by fluorescence microscopy or measurement of the fluorescence in the images (C) (n = 10 for each genotype, p<0.001 by *t*-test). (D) The *rpn-10* mutant also shows an increase in GFP::LGG-1 puncta formation in both the intestine and in the seam cells as shown by fluorescence microscopy. Puncta are indicated by arrows. (E) Scoring of puncta number in the seam cells shows a significant increase in puncta count in the *rpn-10* mutant (n = 41 for WT and = 24 for *rpn-10*, *** represents p<0.0001 by *t-*test). (F) Measurement of puncta number in the intestine also demonstrates a significant increase in puncta number in the *rpn-10* mutant (n = 14 for WT and = 13 for *rpn-10*, *** represents p = 0.0024 by *t*-test). (G) The inhibition of autophagy with *an atg-13* mutation delays the developmental time of an *rpn-10; atg-13* mutant compared to the *rpn-10* or *atg-13* mutants and only 80% of worms reach adulthood (n = 283 for WT, = 298 for *rpn-10*, = 282 for *atg-13*, and = 300 for *rpn-10; atg-13*, p<0.0001 for differences between *atg-13; rpn-10* and *rpn-10* at the 88 hour time point by Fisher’s exact test) (H) The inhibition of autophagy in *rpn-10* mutant worms expressing a Q44::YFP transgene in the intestine through treatment with *atg-13*, *atg-16*.*2*, or *prmt-1* RNAi treatment, starting on day 1 of adulthood, reduces the protective effect of the *rpn-10* mutation. Shown are the percentage of animals with aggregates when scored after 72 hours of RNAi treatment (for *atg-13* RNAi, n = 50, p = 0.0002 by Fisher’s exact test; for *atg-16*.*2* RNAi, n = 50, p = 0.003; and *prmt-1* RNAi, n = 50, NS).

The ability of *skn-1* to independently control oxidative stress response genes and proteasome subunit expression suggested that additional transcription factors could act in parallel to *skn-1* and contribute to this specificity. To identify such transcription factors, we screened two separate RNAi libraries consisting of subsets of transcription factors drawn from the Ahringer and Vidal RNAi libraries for clones that produced developmental phenotypes in the *rpn-10* mutant that are similar to those produced by *skn-1* RNAi (S6 Table). From the two independent screens, we identified *elt-2*, which is a GATA transcription factor and is essential for the expression of most genes expressed in the intestine [76–78]. In addition to its developmental role, *elt-2* is required for innate immunity, contributes to the response to heavy metal exposure, and contributes to the beneficial effects of changes in *daf-2*/IGFR signaling on worm lifespan [79–82]. In the *rpn-10* mutant, we found that inhibiting *elt-2* with RNAi mirrored the effects of *skn-1* knock-down and produced small, sickly, animals that often arrested during larval development (Fig 6A).

To determine if *elt-2* cooperates *with skn-1* to control the expression of either oxidative stress response genes or proteasome subunit genes in the *rpn-10* mutant, we used Nanostring analysis to measure the expression of these genes in *rpn-10* mutants treated with control or *elt-2* RNAi. In contrast to *skn-1* RNAi, we found that *elt-2* RNAi did not block the activation of either group of genes (Fig 5D and 5E, and S5 Table). Hence, while *elt-2* is similarly required for the viability of the *rpn-10* mutant, *elt-2* likely acts via a distinct mechanism than *skn-1*, and is discussed further below.

### Autophagy is activated in the *rpn-10* mutant

As discussed previously, our RNAi-seq studies identified the autophagy genes *atg-16*.*2* and *prmt-1/epg-11* as being up-regulated in the *rpn-10* mutant (S2 and S4 Tables). This observation is consistent with the activation of autophagy observed when proteasome function is potently reduced via the expression of a dominant-negative proteasome subunit or is chronically reduced in cultured neural cells by long-term exposure to proteasome inhibitors [83, 84]. To determine if autophagy is activated in the *rpn-10* mutant, we examined the expression and subcellular localization of the LGG-1 protein in wild-type and *rpn-10* mutant animals via the use of a transgene expressing a GFP::LGG-1 fusion protein [85]. The LGG-1 protein is the worm ortholog of LC3 and has been shown to play an analogous role in the formation of autophagosomes via integration into the autophagosome membrane [85]. We initially found that the expression of the GFP::LGG-1 fusion protein is increased in the *rpn-10* mutant compared to wild-type animals (Fig 6B and 6C), and this increase in *lgg-1* expression occurs in part at the transcriptional level and in conjunction with the transcriptional up-regulation of the worm beclin ortholog *bec-1* (S8 Fig) [85]. The up-regulation of LGG-1 expression has also been seen in worms with activated autophagy resulting from either removal of the germline via a *glp-1* mutation or the inhibition of *let-363*/TOR with *let-363* RNAi treatment [86]. Based on this and other work, the increased expression of LGG-1 has been therefore suggested to indicate the activation of autophagy [87]. To seek additional evidence of enhanced autophagy in the *rpn-10* mutant, we looked for the presence of GFP::LGG-1 puncta, which are produced by the integration of LGG-1 into the membrane of developing autophagosomes [85]. We introduced a GFP::LGG-1 reporter into the *rpn-10* mutant and observed the effects in the intestine and seam cells of the wild-type and *rpn-10* mutant transgenic animals [86]. We saw an increase in GFP-positive puncta in both the seam cells and intestine of the *rpn-10* mutant animals compared to the wild-type controls, which suggests an increase of the activity of the autophagy-lysosome pathway in these animals (Fig 6D, 6E and 6F).

### The activity of the autophagy pathway is essential for the development and enhanced proteostasis of the *rpn-10* mutant animals

The increase in autophagy could be involved in the adaptation to the changes in UPS activity in the *rpn-10* mutant and could also contribute to some aspect of the beneficial effects of this mutation on proteostasis. To examine these possibilities we tested the effects of autophagy inhibition on both the development and improved proteostasis of the *rpn-10* mutant. For these studies we focused on the *epg-1*/*atg-13*, *prmt-1/epg-11*, and *atg-16*.*2* genes based upon either their identification in our RNA-seq studies (S2 Table) or identified role in the clearance of protein aggregates via autophagy [70, 88]. During development, we found that loss of the *atg-13* gene greatly impaired the development of the *rpn-10* mutant as exhibited by the significantly delayed development of an *rpn-10; atg-13* mutant compared to either mutant alone (Fig 6G). Notably, almost 20% of the *rpn-10; atg-13* mutants appeared to be permanently arrested during development and failed to reach adulthood even after five days (Fig 6G).

We also tested the role of autophagy in the enhanced resistance to the accumulation of polyglutamine-repeat protein aggregates in the intestine of animals expressing a Q44::YFP transgene by inhibiting the *prmt-1/epg-11*, *atg-16*.*2*, and *atg-13* genes via the use of RNAi starting on day 1 of adulthood. We began RNAi treatment at this time point to prevent any adverse effects of RNAi treatment from occurring during development. We found that the knock-down of either *atg-13* or *atg-16*.*2* produced an increase in the percentage of animals with aggregates compared to the control RNAi treated *rpn-10* mutant animals (Fig 6H). In contrast, these RNAi treatments only modestly increased the percentage of wild-type animals with aggregates (S9 Fig). To further explore the effects of these RNAi treatments, we also counted the number of aggregates in each worm in a separate trial. We again found that *atg-13* and *atg-16*.*2* RNAi treatment had a greater effect on the *rpn-10* mutants compared to the wild-type animals, and additionally now *prmt-1* RNAi produced a selective increase in polyglutamine aggregation in the *rpn-10* mutant (S9 Fig). Together these findings show a vital role for autophagy in both promoting the normal development of the *rpn-10* mutant animals, and contributing to the effects of the *rpn-10* mutation on proteostasis.

### Lysosome function is essential for the development of the *rpn-10* mutant

**Fig 7:**
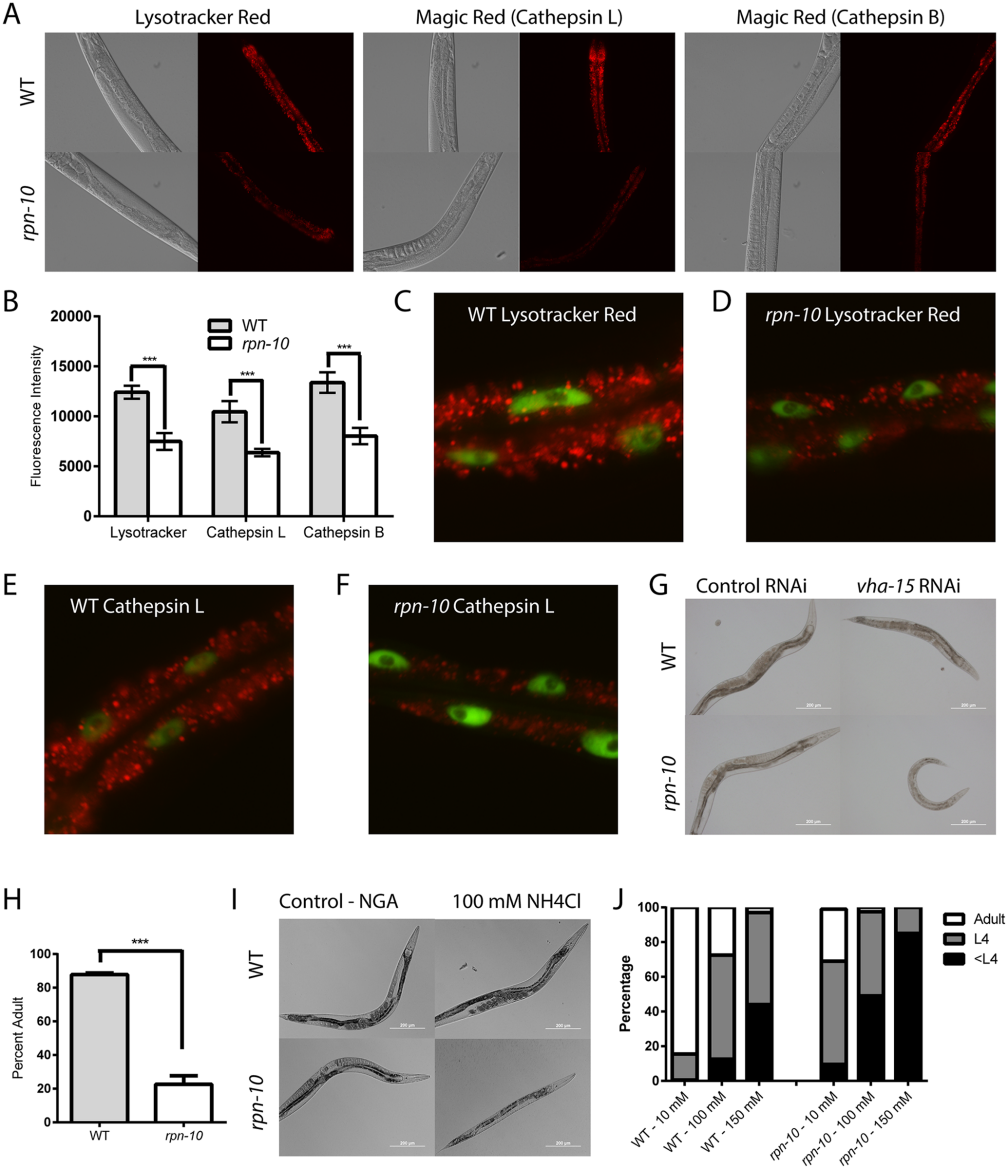
Lysosome function is required for the viability of the *rpn-10* mutant. (A) Representative images of day 1 adult wild-type and *rpn-10* mutant animals stained either with Lysotracker red, which accumulates in lysosomes due to the low pH environment of the organelle, or with Magic Red substrates that produce red fluorescence following proteolytic cleavage by cathepsin L or cathepsin B, respectively. (B) Quantification of the red fluorescence in images captured as in panel A (n > 9 for all genotypes, p = 0.0001 for WT vs *rpn-10* for Lysotracker red, = 0.0005 for Magic Red B, and = 0.0007 for Magic Red L). (C and D) High-magnification images of Lysotracker red stained animals, wild-type in C and *rpn-10* in D, captured as in panel A to demonstrate details of the intestine. GFP fluorescence indicates the intestinal nuclei as marked by an *elt-2p*::*GFP* reporter gene. (E and F) Images of animals stained with the Magic Red dye specific for cathepsin L, which are cropped similarly to panels C and D; GFP fluorescence indicates the intestinal nuclei. (G) Images showing the effects of treating wild-type and *rpn-10* mutant animals with *vha-15* RNAi. (H) Graph quantifying the effects of *vha-15* RNAi treatment on wild-type and *rpn-10* mutant animals (*** represents p = 0.0062 by *t-*test). (I) Images showing the developmental delay produced by treating the *rpn-10* mutant with 100 mM NH_4_Cl to neutralize lysosomal pH. (J) Graph showing the developmental effects of increasing NH_4_Cl doses on wild-type and *rpn-10* mutant animals.

The increased autophagic activity seen in the *rpn-10* mutant could act to shuttle proteins to the lysosome for degradation via lysosomal proteases. To examine the role of the lysosomes in the *rpn-10* mutant, we visualized the intestinal lysosomes through staining with both the Lysotracker fluorescent dye, which concentrates in lysosomes due to their low pH, and the use of the Magic Red cathepsin B and cathepsin L substrates [89]. The cathepsin B and L substrates are cell-permeable cresyl violet-conjugated peptides containing either the Arg-Arg or Phe-Arg sequence cleaved by the respective cathepsin inside of the lysosome. These cleavage events relieve the intramolecular quenching of the cresyl violet fluorophore and produces red fluorescence. Our work represents the first application of the Magic Red substrates in *C*. *elegans* research as a novel approach to identify lysosomes and quantify their activity. The use of the cathepsin substrates was particularly attractive because the location and degree of fluorescence are directly related to the activity of the cathepsin enzymes [89]. With the Lysotracker dye, we saw staining of intestinal lysosomes, and a similar pattern was observed when the animals were stained with either the cathepsin B or cathepsin L substrates (Fig 7A). Consistent with both Lysotracker and the Magic Red substrates acting to label lysosomes, we observed a high-degree of co-localization when wild-type animals were stained with both Lysosensor Green and the cathepsin B substrate, as evidenced by Pearson’s correlation score or Mander’s overlap score which averaged greater than 0.9 (Pearson average 0.92, n = 15 and Mander average 0.94, n = 15)(S10 Fig) [90].

When the staining of the wild-type and *rpn-10* mutant animals were compared, we observed an overall decline in both lysosome volume and in the activity of each of the cathepsins as evidenced by reduced fluorescence in the mutant (Fig 7A and 7B). The decline in overall fluorescence was likely due to a reduction in lysosome number and volume in the *rpn-10* mutant (Fig 7C, 7D, 7E and 7F). Together our observations could suggest that the cellular lysosome pool is being consumed by an increase in autophagy via the fusion of the lysosomes with the enlarged pool of autophagosomes.

To test the importance of lysosome function in the *rpn-10* mutant, we treated worms with RNAi to inhibit *vha-15*, which is a part of the vacuolar proton-translocating ATPase and acts to promote the acidification of the lysosomes [91]. If the *rpn-10* mutant relied upon the autophagosome-lysosome pathway to compensate for the declines in proteasome activity, we expected these mutants to show enhanced sensitivity to lysosome inhibition with *vha-15* RNAi compared to wild-type animals. Consistently, we found that the *rpn-10* mutant shows a decrease in body size and developmental rate compared to N2 animals treated in parallel with *vha-15* RNAi (Fig 7G and 7H). To determine if these effects could result from changes in lysosome pH, we treated worms with NH_4_Cl which accumulates in lysosomes and neutralizes the normally acidic pH of the organelle, producing a decrease in proteolytic activity [92]. Consistent with the effects of *vha-15* RNAi, we observed the *rpn-10* mutant animals to develop slowly following treatment with increasing concentrations of ammonium chloride, while N2 worms treated in parallel showed a lesser effect (Fig 7I and 7J). These data suggest that the *rpn-10* mutant both exhibits an increase in autophagy and has become dependent on the activity of the autophagy-lysosome pathway for normal development as evidenced by the selective vulnerability of the mutant to lysosome inhibitors.

### *elt-2* controls lysosome formation in the gut

**Fig 8:**
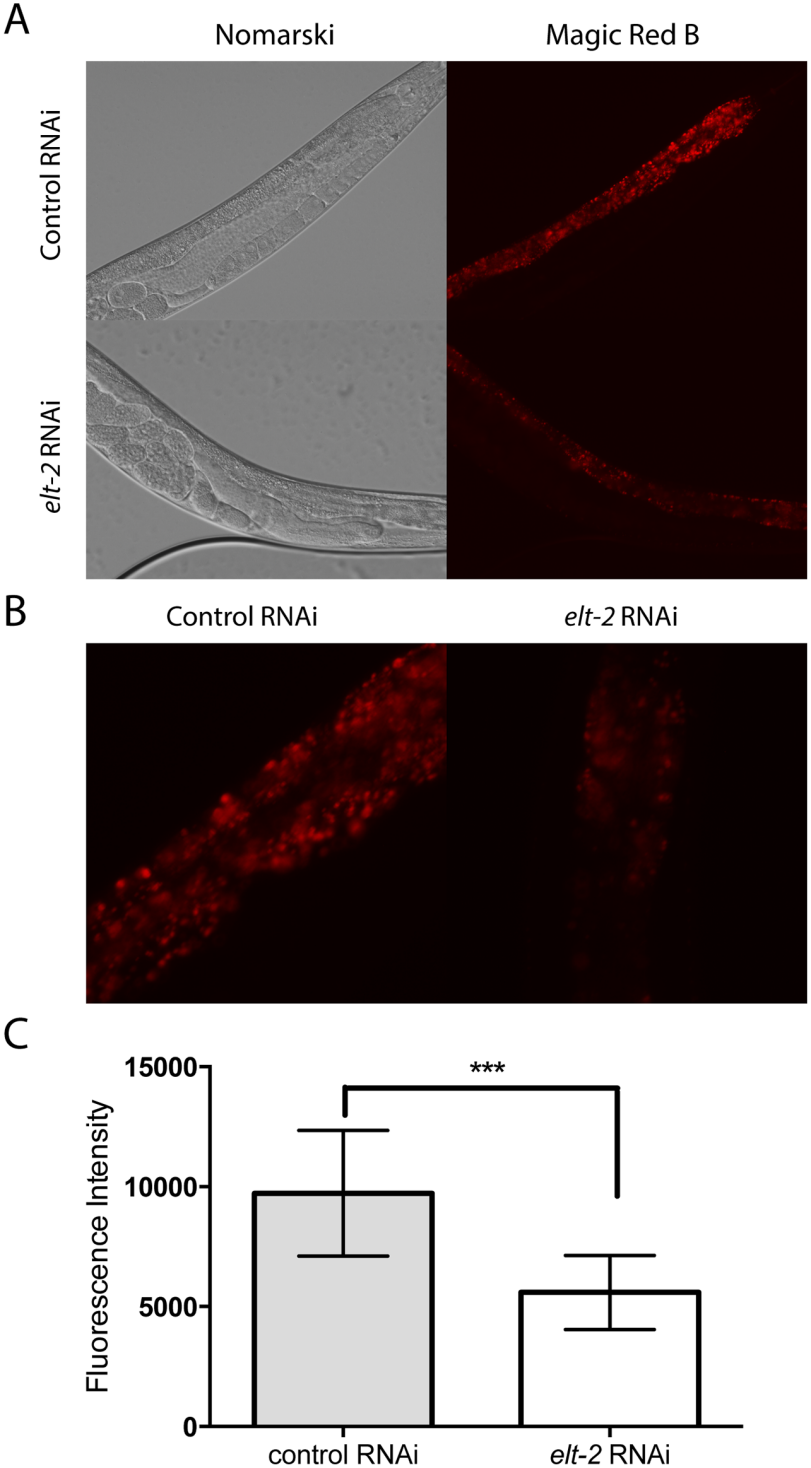
*elt-2* controls lysosome formation in the intestine. (A) Representative images of day 1 adult animals treated with control or *elt-2* RNAi and stained with the Magic Red dye specific for cathepsin B. (B) Images of animals captured as in panel A, but cropped instead of reduced in scale, to demonstrate details of the intestine. (C) Graph quantifying the effects of control and *elt-2* RNAi on Magic Red fluorescence (n = 15 for each RNAi treatment, p < 0.001 by *t-*test).

To identify the pathway that could be controlled by *elt-2* and contribute to the survival of the *rpn-10* mutant, we explored whether *elt-2* might be involved in the generation of lysosomes in the intestine. Initially, we utilized an existing gene expression dataset which was generated using Serial Analysis of Gene Expression, SAGE, to compare gene expression differences in RNA prepared from L1 larvae that either lacked *elt-2* due to the *elt-2(ca15)* mutation or contained the mutation as well as a rescuing transgene expressing *elt-2* [77]. In this dataset, we noted that the expression of multiple genes associated with lysosomes, including the lysosome membrane protein *lmp-1*, vacuolar proton-translocating ATPase subunits, and cathepsins, all showed decreased expression in the *elt-2(ca15)* mutants compared to the *elt-2*^+^ larvae (Table 1). To determine if these changes in gene expression affected lysosome size, number, or function, we stained worms grown on control RNAi or *elt-2* RNAi with the Magic Red cathepsin B substrate. This staining demonstrated a reduction in both fluorescence intensity and the number of lysosomes present in the RNAi treated worms (Fig 8A, 8B and 8C). The results are consistent with either a reduction in lysosome production or a reduction in the proteolytic activity of the lysosome following *elt-2* RNAi treatment.

**Table 1:** Control of lysosome gene expression by *elt-2*.

Gene	Ratio *elt-2*^+^/*elt-2(ca15)*
*lmp-1*	2.1
*vha-3*	236.0
*vha-6*	20.7
*vha-11*	1.6
*vha-14*	2.4
*vha-16*	1.8
*cpr-1*	8.0
*cpr-2*	64.0
*cpr-3*	6.1
*cpr-5*	1278.0
*cpr-6*	138.1

Ratio of gene sequence counts detected in L1 larval animals either expressing ELT-2 from a transgene (*elt-2*^+^) or lacking *elt-2* due to a genetic mutation (*elt-2*^null^). For genes showing a zero count in the *elt-2*^null^ condition, a value of 0.1 was used to avoid an error. Data was obtained from the supplemental materials of McGhee, et. al. [77].

### *elt-2* and *skn-1* are limiting in the *rpn-10* mutant

**Fig 9:**
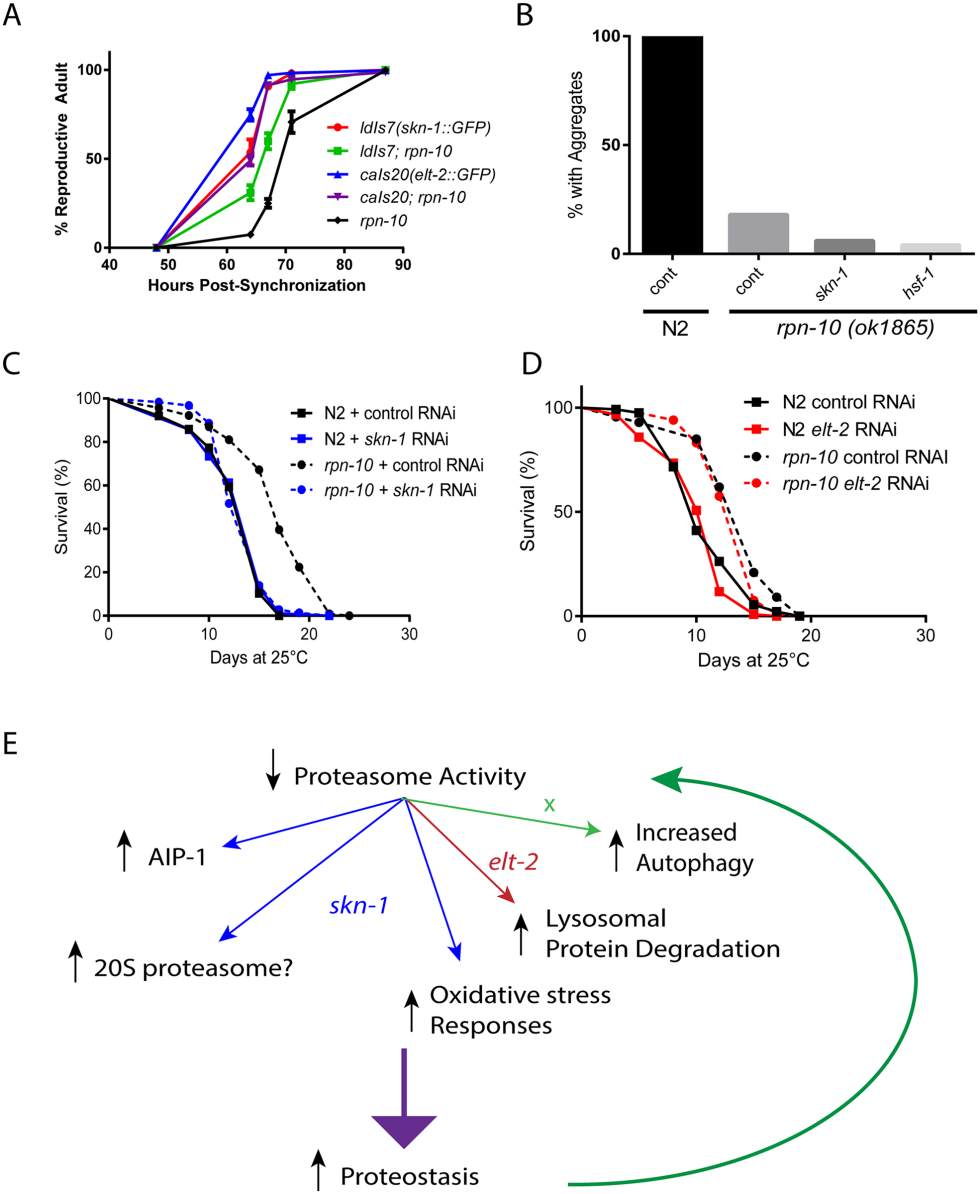
Roles of *skn-1* and *elt-2* in the development, lifespan, and enhanced proteostasis of the *rpn-10* mutant. (A) The *ldIs7* and *caIs20* transgenes express either *skn-1* or *elt-2* as a GFP fusion protein under the control of the native promoter. Each transgene was crossed into the *rpn-10* mutant, which also has the endogenous *skn-1* or *elt-2* genes intact. Development of synchronized *rpn-10* and transgenic worms was measured and plotted as shown. (B) *rpn-10* mutant worms treated with *skn-1* or *hsf-1* RNAi starting at day 1 of adulthood did not show an increase in Q44::YFP aggregation in the intestine (n = 50 for WT and *rpn-10*). (C) Treatment with *skn-1* RNAi mitigates the extended lifespan phenotype seen in the *rpn-10* mutant when kept at 25°C from day 1 of adulthood. Mean lifespan for N2 on control RNAi is 13.0 days (n = 102) and 13.3 days on *skn-1* RNAi (n = 119). Mean lifespan for *rpn-10* on control RNAi is 16.7 days (n = 116) and is 13.8 days on *skn-1* RNAi (n = 126). Comparison of *rpn-10* mutant treated with control vs *skn-1* RNAi, p<0.0001 by log-rank test; comparison of N2 and *rpn-10* on control RNAi, p<0.0001 by log-rank test. (D) Treatment with *elt-2* RNAi has no effect on the extended lifespan of the *rpn-10* mutant at 25°C. Mean lifespan for N2 on control RNAi is 11.1 (n = 120) and is 10.4 days on *elt-2* RNAi (n = 113). Mean lifespan for *rpn-10* on control RNAi is 13.7 days (n = 113) and is 13.3 days on *elt-2* RNAi (n = 119). Comparison of *rpn-10* mutant treated with control vs *elt-2* RNAi, p<0.03 by log-rank test; comparison of N2 and *rpn-10* on control RNAi, p<0.0001 by log-rank test. (E) Model summarizing the effects of *skn-1* and *elt-2* in the *rpn-10* mutant and possibly other situations where proteasome function is reduced.

While the inhibition of either *elt-2* or *skn-1* in the *rpn-10* mutant is harmful, it was less clear to what extent either transcription factor normally acts to promote the growth and survival of the *rpn-10* mutant. To determine if enhancing the function of either gene is beneficial, we used transgenes to over-express either *elt-2* or *skn-1* in the *rpn-10* mutant. During the construction of these strains, we observed that the transgenic animals appeared to develop and reproduce faster than the non-transgenic worms. To directly test whether the development of the *rpn-10* mutant was at least somewhat normalized by the over-expression of either gene, we performed development assays on synchronized animals using successfully reaching reproductive adulthood as the outcome. We found that the over-expression of either transcription factor led to more rapid development with *elt-2* over-expression perhaps having a somewhat stronger effect than *skn-1* (Fig 9A). This finding suggests that the activities of *elt-2* and *skn-1* are limiting in the *rpn-10* mutant and likely contribute to the slight developmental delay exhibited by these animals. Hence, enhancing the activity of either transcription factor leads to the increased activity of downstream targets and enhances the development of the *rpn-10* mutant.

### Complex roles of *elt-2* and *skn-1* in the beneficial effects of the *rpn-10* mutation

While both *elt-2* and *skn-1* both contribute to the development of the *rpn-10* mutant, it is unclear if either contribute to the improved proteostasis and increased longevity exhibited by the *rpn-10* mutant. We first tested whether the inhibition of *skn-1*, *elt-2*, or *hsf-1* affected the reduction in Q44::YFP aggregates seen in the *rpn-10* mutant animals by using RNAi to knock-down each gene only in adult animals. We took this approach due to the adverse effects that both *elt-2* and *skn-1* had during development. We found that neither *skn-1* nor *hsf-1* was required for the reduction in protein aggregates (Fig 9B). The Q44::YFP transgene was controlled by the *vha-6* promoter, and this promoter appears to be regulated by *elt-2* because we observed a dramatic decline in YFP fluorescence in either wild-type or *rpn-10* mutant transgenic animals treated with *elt-2* RNAi. We then examined the role of *skn-1* and *elt-2* in the increased longevity exhibited by the *rpn-10* mutant by treating adult wild-type or *rpn-10* mutant worms with control, *skn-1*, or *elt-2* RNAi and then measuring the effects on lifespan. We found that the increased lifespan of the *rpn-10* mutant required *skn-1* but not *elt-2* (Fig 9C and 9D). Lastly we examined the role of *skn-1* in the enhanced oxidative stress resistance exhibited by the *rpn-10* mutant. We were unable to obtain adult *rpn-10* mutant animals following treatment with *skn-1* RNAi, so we shifted to using larval animals that had been treated with control or *skn-1* RNAi for 48 hours. Treatment of these animals with 100 μM juglone revealed that the *skn-1* RNAi treatment markedly reduced the oxidative stress resistance of the *rpn-10* mutant (S11 Fig). Together these data suggest that *skn-1* is essential for development, oxidative stress resistance, and longevity but not improved proteostasis of the *rpn-10* mutant, while *elt-2* is only essential for development. The role of *elt-2* in proteostasis is difficult to judge since many promoters active in the intestine are *elt-2* target genes [78].

## Discussion

### Loss of *rpn-10* produces proteasome dysfunction

The inhibition of the majority of the proteasome subunits in *C*. *elegans* is clearly detrimental, resulting in phenotypes such as developmental arrest or a marked reduction in body size [26]. Similarly, essentially all of the proteasome subunits with deletion alleles are homozygous lethal and need to be maintained as a balanced heterozygote (see Wormbase for details). Therefore, it was somewhat surprising that two independent viable deletion alleles for *rpn-10* have been isolated. This could have been due to the expression of *rpn-10* in a limited pattern in the animal, or the presence of a redundant subunit. Instead, we find that *rpn-10* is broadly expressed and produces aspects of proteasome dysfunction in multiple tissues when removed. Despite these findings, the viability of this *rpn-10* deletion mutant could imply that the worm proteasome is similar to those of yeast, which are also less dependent on the analogous Mcb1/RPN10 subunit, or that sufficient compensatory pathways can be activated in the worm *rpn-10* mutant to cope with the reductions in proteasome activity [29]. Our work suggests that the activation of compensatory mechanisms, such as the up-regulation of other proteasome subunits via the actions of *skn-1* and the increased use of autophagy and lysosomes possibly for protein degradation are an important aspect of the response to the chronic reduction in proteasome activity seen in the *rpn-10* mutant (Fig 9E). Within this response *elt-2* may play a supportive role by maintaining an adequate pool of intestinal lysosomes (Fig 9E). The pathway leading to changes in the expression of autophagy genes and autophagic activity are currently unclear but recent work has shown that the RPN-10 protein serves as an adapter to facilitate the clearance of the proteasome via autophagy in *Arabidopsis* [93]. Perhaps the loss of RPN-10 could stimulate autophagic activity via the loss of some form of negative feedback from the proteasome. Alternately, the 19S cap proteasome subunits have been shown to dissociate from the 20S subunit in the setting of proteasome dysfunction and then bind to protein aggregates. When localized to the aggregates, the deubiquitinase activity of RPN-11/Poh1 releases ubiquitin chains from the aggregated protein, and these ubiquitin chains then serve to activate autophagy via the HDAC6 protein [94, 95]. It could be possible that the loss of RPN-10 destabilizes the 26S proteasome or via other mechanisms promotes the association of RPN-11 and other subunits with cellular protein aggregates to then promote their removal via autophagy. Finally, proteomic studies comparing wild-type and long-lived *daf-2* mutants in *C*. *elegans* have made the unexpected finding that the *daf-2* mutants have increased amounts of insoluble protein during aging compared to wild-type animals [96]. Further, the insoluble fraction includes increased amounts of small heat-shock proteins, which could suggest that the controlled aggregation of proteins is an important mechanism for enhancing proteostasis. Perhaps, the *rpn-10* mutant might act in a similar manner, especially in light of the enhanced heat-shock response observed, with the shunting of UPS substrates into a protected insoluble protein compartment in the cell. Future work could test this possibility via the analysis of amounts and types of insoluble proteins present in the *rpn-10* mutant.

However, it is also possible that the spectrum of UPS substrates that fail to be degraded in the *rpn-10* mutant differs in an important way from those that accumulate when other subunits are removed via RNAi or genetic mutations. In the Mcb1/RPN10 yeast mutant, UPS substrates that are degraded via the N-end rule pathway are degraded to a similar extent as seen in wild-type yeast, whereas the substrates degraded via the ubiquitin fusion pathway are no longer degraded normally [29]. These differential effects on substrate degradation could reflect the presence of multiple ubiquitin binding proteins in the 19S proteasome subunit or the ability of the 20S proteasome to independently degrade some UPS substrates [30, 31, 97, 98]. Future work can explore this question via the use of proteomic approaches, such as ubiquitin-remnant profiling, to determine if differences in substrate degradation are also observed in *C*. *elegans*. In addition, these experiments could determine the identities of substrates that might account for the phenotypic differences observed between the *rpn-10* mutant and mutations or RNAi affecting other subunits [66, 99, 100].

### Graded proteasome dysfunction can have beneficial effects on proteostasis and longevity

An unexpected finding in our work was the increased lifespan and improved responses of the *rpn-10* mutant to proteostasis threats including heat, oxidative stress, and the expression of metastable, unstable or aggregation-prone proteins. Based on prior work in the field, we hypothesized that these animals would be sensitive to proteostasis threats [38–46]. However, these previous experiments generally relied upon potent inhibition of proteasome activity via the use of chemical inhibitors or RNAi approaches, so there may be important differences in proteasome activity between the *rpn-10* mutant and these other interventions, or in the types of compensatory pathways involved in the response to each. Furthermore, our findings are consistent with prior work showing that either cultured cells or yeast exposed to proteasome inhibitors show elevated expression of heat shock proteins, and increased resistance to heat shock [47, 101, 102]. We also find that genes involved in the response to oxidative stress are up-regulated in the *rpn-10* mutant so these two compensatory responses may contribute to the ability of the *rpn-10* mutant to better survive the exposure to heat or oxidative stress.

The reasons accounting for the improved ability of the *rpn-10* mutant to prevent the aggregation of the unstable polyglutamine repeat proteins are still somewhat unclear. Data from vertebrate cells suggest that proteasome inhibition may occur prior to the development of polyglutamine protein aggregates, so some aspect of the compensation to proteasome dysfunction, as opposed to proteasome activity alone, may play a key role in determining the timing and levels of protein aggregation [103]. In addition to the UPS, autophagy has been identified as an important pathway for the degradation of polyglutamine repeat proteins. Consequently a cellular environment where there is a high level of autophagic activity may result in low levels of protein aggregation regardless of the level of UPS activity [104]. Our data suggest that this could be at least partially true, because we find that the increased flux in the autophagosome-lysosome pathway in the *rpn-10* mutant contributes to at least some of the reduction in aggregation by facilitating the removal of the polyglutamine repeat proteins. Alternately, the increased expression of oxidative stress response genes and priming of the heat-shock response which occur in the *rpn-10* mutant could also act to reduce protein mis-folding and aggregation.

### *skn-1* and *elt-2* are required to survive proteasome dysfunction

An important response to the reduction in UPS activity in the *rpn-10* mutant is the activation of the *skn-1*/Nrf2 transcription factor which then promotes the expression of proteasome subunits, and accessory factors like *aip-1*/AIRAP, as part of a “bounce-back” response [22, 25, 26]. Importantly we find that the *rpn-10* mutant not only uses this response, but also actually requires the continuous activity of this response in order to develop normally and survive long-term in the presence of chronic reductions in proteasome activity. We also identify a novel role for the *elt-2* GATA transcription factor in promoting the survival of the *rpn-10* mutant despite the presence of chronic proteasome dysfunction. This effect could occur through a basal role in determining the level and activity of lysosomes in the worm intestine, or *elt-2* activity could somehow be enhanced in the setting of proteasome dysfunction. Consistently, *elt-2* has been identified as a UPS substrate, and the UPS-mediated degradation of ELT-2 contributes to the killing of worms by the bacterial pathogen *Burkholderia pseudomallei* [105]. Furthermore, the vertebrate GATA-1 and GATA-2 transcription factors are known UPS substrates [106–108]. Under normal conditions, GATA-2 is usually rapidly degraded by the UPS, and this event determines the ratio of GATA-1 and GATA-2 bound to target gene promoters [106–108]. In worms, we failed to observe significant changes in the expression or localization of an ELT-2::GFP fusion protein in the *rpn-10* mutant, but *elt-2* activity could be altered in other ways in response to changes in UPS activity, such as through post-translational modifications or associations with specific binding partners.

### Could similar compensatory pathways also be activated when proteasome subunits are over-expressed?

Recently several groups have shown that the elevated expression of specific proteasome subunits leads to increased proteostasis and enhanced longevity in both *Drosophila* and *C*. *elegans* [18, 56, 98, 109]. However, one recent study demonstrated that the expression of the over-expression of a single subunit, *pbs-5*, in *C*. *elegans* with a transgene unexpectedly also leads to the increased expression of the endogenous genes encoding other proteasome subunits [98]. Furthermore, the over-expression of *pbs-5* leads to the increased expression of the *gst-4p*::*GFP* reporter, suggesting an increase in *skn-1* activity in this mutant. Consistent with a role for *skn-1* acting downstream of the increase in PBS-5 expression, the increased lifespan observed in these transgenic worms required the activity of *skn-1* [98]. One possible model to account for both the requirement for *skn-1* and the elevated expression of non-transgene encoded proteasome subunits would be for the initial imbalanced expression of PBS-5 to disrupt rather than enhance the assembly of active proteasomes, which could trigger at least some of the compensatory mechanisms also utilized by the *rpn-10* mutant in an effort to promote the formation of active proteasomes and maintain proteostasis. The authors examined the expression of proteasome subunit genes following the treatment of the transgenic worms with *skn-1* RNAi and did see small, but non-significant decreases in subunit expression [98]. These findings could suggest that *skn-1* only plays a minor role in controlling proteasome subunit expression following increased PBS-5 expression, and that the expression of other *skn-1* target genes, such as the oxidative stress response or other novel pathways, may be more important in an aging context. It will be interesting to explore whether *elt-2*, autophagy, or lysosome activity play roles in the effects of this transgene on stress resistance and aging phenotypes. If so, our work using the *rpn-10* mutant could identify molecular pathways that could be exploited to improve proteostasis via either the augmentation or graded inhibition of proteasome activity.

## Materials and Methods

### Strains

The strains CB1157 (*unc-54(e1157)*), CL2166 (*dvIs19[pAF15 (gst-4*::*GFP*::*NLS)]*) [53], CL2070 (*dvIs70[hsp-16*.*2*::*GFP; rol-6 (su1006)]*) [54], DA2123 (*adIs2122 [lgg-1p*::*GFP*::*lgg-1 + rol-6(su1006)]*) [110], HZ1688 (*atg-13(bp414)*) [88], MAH236 (*sqIs13 [lgg-1p*::*GFP*::*lgg-1 + odr-1p*::*RFP]*) [86], SJ4001 (*zcIs1[aip-1*::*GFP]*) [37], and VC1369 (*rpn-10(ok1865)*) were obtained from the *Caenorhabditis* Genetics Center which is funded by NIH Office of Research Infrastructure Programs (P40 OD010440). The *rrIs1[elt-2*::*lacZ*::*GFP]* transgene which expresses nuclear localized GFP in the intestine has been previously described [111]. The *rpn-10(ok1865)* mutant was outcrossed against N2 five times, and one resulting outcrossed homozygous line was used for all subsequent crosses and experiments. The presence of the *rpn-10(ok1865)* allele was determined by single-worm PCR using oligonucleotides designed to amplify the wild-type *rpn-10* allele (F 5’-AAGAGAACAACGCGCATCTT-3’; R 5’-GTGTGCCCCTTTGAGGAGTA-3’) and to detect the deletion present in the *rpn-10(ok1865)* allele (F 5’-CCCATTCCAATTGTTGCTCT-3’; R 5’-TGCACCAACAACTCCACATT-3’). The strain AM140 (*rmIs132[unc-54p*::*Q35*::*YFP]*) was kindly provided by Dr. Richard Morimoto and previously described by our group [26, 112]. The strain AM446 (*rmIs223[phsp70*::*gfp]; pRF4[rol-6(su1006)]*) was kindly provided by Dr. Richard Morimoto [55]. The strain PP608 *(hhIs64[unc-119(+); sur-5*::*UbV-GFP]; hhIs73[unc-119(+); sur-5*::*mCherry])* was kindly provided by Dr. Thorsten Hoppe [36]. The strain BC14890 (*sIs14010[rpn-7*::*GFP]*) was kindly provided by Dr. David Baillie [113]. The strain OG412 (*drIs20[vha-6p*::*Q44*::*YFP + rol-6(su1006)]*) was kindly provided by Dr. Todd Lamitina [48]. The strain JM168 (*elt-2(ca15); caIs20[elt-2p*::*elt-2*::*GFP* + *unc-119(+)]*) was kindly provided by Dr. James McGhee. This strain was produced by gamma irradiation of JM73, which carried the transgene as an extrachromosomal array, to integrate the array into the genome followed by backcrossing to remove extraneous mutations [114]. Strains containing transgenes, genetic mutations, and the *rpn-10(ok1865)* mutation were generated by standard crossing and genotyped by PCR.

### Transgenic animals

Worms expressing a RPN-10::GFP fusion protein were generated via biolistic bombardment [115]. The 6236103120536928 H12 fosmid clone which contains the entire *rpn-10* coding sequence in fosmid WRM0618DC02 fused to GFP at the C-terminus as well as >10 kilobases of 5’ and 3’ flanking sequences was requested from the TransgenOme project, and the presence and location of the GFP insert was confirmed by PCR and sequencing [33]. The fosmid was purified from *E*. *coli* and used to generate transgenic animals via bombardment using the DP38 (*unc-119(ed3)*) strain as previously described [115, 116]. Transgenic animals were identified via rescue of the mobility and body size defects of the *unc-119* mutant. This resulted in the isolation of the ALF85 (*bafEx85*) transgenic strain, which was outcrossed with N2 and then used for further study.

### RNAi treatment

The *hsf-1*, *pas-6*, *pbs-6*, *pbs-7*, *rpn-12*, *skn-1*, and *wdr-23* RNAi clones were previously described [26]. The *atg-13*, *atg-16*.*2*, *elt-2*, *prmt-1*, vha*-15*, and *rpn-10* RNAi clones were retrieved from the Ahringer RNAi library and confirmed by sequencing [117]. For RNAi treatment, NGA plates containing 50μg/ml carbenicillin and 0.2% β-lactose (in place of IPTG for dsRNA induction) were spotted with overnight cultures of RNAi bacteria inoculated from discrete individual colonies [118]. Due to the adverse developmental effects of the *elt-2* and *vha-15* RNAi, these clones were typically diluted 1:10 with bacteria containing the empty vector control RNAi clone prior to spotting on the plates. Unless otherwise noted, eggs isolated by hypochlorite treatment were then placed on spotted RNAi plates and incubated at 20°C.

### Fluorescence microscopy with GFP reporters

Approximately 50–100 eggs isolated via hypochlorite treatment were placed on NGA plates spotted with *E*. *coli* strain OP50-1 and grown to adulthood at 20°C. Digital images of day 1 adult worms were either captured with an Olympus BX51 upright microscope and DP70 camera as previously described or with a Nikon Eclipse Ti inverted microscope with a 14-bit CoolSNAP HQ2 (Photometrics) CCD camera and Nikon Elements software [26, 119]. Fluorescence intensity of the respective reporters was then quantified using ImageJ and statistical analysis of the resulting image data was completed in Prism6 (GraphPad Software, San Diego, CA) [120].

To perform heat-shock studies with the *hsp-16*::*GFP* and *hsp-70*::*GFP* reporters, approximately 20 day 1 adult animals drawn from the same synchronized populations used for the initial baseline imaging were transferred to fresh, spotted NGA plates. These worms were incubated at 35°C for one hour and allowed to recover at 20°C overnight (approximately 14 hours) before capturing the post-heat shock images.

To assess effects of the *rpn-10* mutation on GFP::LGG-1 expression the *adIs2122 [lgg-1p*::*GFP*::*lgg-1 + rol-6(su1006)]* transgene was outcrossed into N2 and *rpn-10* mutant animals via standard crosses. Synchronized day 1 adult animals were mounted and GFP fluorescence was measured via the analysis of digital images with ImageJ. To assess changes in autophagic activity, the *sqIs13 [lgg-1p*::*GFP*::*lgg-1 + odr-1p*::*RFP]* transgene was outcrossed into N2 and *rpn-10* mutant animals via standard crosses. Synchronized day 1 adult animals were mounted and the animals were photographed using a GFP filter set. GFP::LGG-1 puncta in individual seam cells were counted and then analyzed for mean and statistical significance using Prism6 (GraphPad Software, San Diego, CA). GFP::LGG-1 puncta in the intestine were counted by using the “Find Maxima” function in ImageJ, and the puncta counts were then analyzed for mean and statistical significance using Prism6.

### Lifespan assays

Lifespan assays were conducted at 25°C using either NGA or RNAi plates containing 50 μM FUDR as previously described [119]. All worms were synchronized by hypochlorite treatment, hatched, and grown to adulthood at 20°C on NGA plates supplemented with streptomycin (0.2 mg/mL) and spotted with *E*. *coli* strain OP50-1. They were transferred to plates containing FUDR (and RNAi when appropriate) on the first day of adulthood and placed at 25°C. The worms were transferred to a second FUDR plate on the second day and left at 25°C for the remainder of the assay. Lifespan assays without RNAi treatment were conducted on NGA plates containing streptomycin (0.2 mg/mL) as well as FUDR (50 μM) and spotted with OP50-1. RNAi treatment lifespans were conducted on NGA plates supplemented with FUDR, carbenicillin (50 mg/mL), and isopropyl β-d-thiogalactopyranoside (IPTG,1 mM). These plates were spotted with OP50(xu363) bacteria, which is an OP50-derived bacterial strain that can deliver RNAi to worms, that had been transformed with RNAi-expressing plasmids [121]. Prism6 (Graphpad Software) was used to generate graphs and perform log-rank testing for curve comparisons. STATA 8 was used to create lifetables and calculate mean survival.

### Developmental assays

The worms were synchronized by hypochlorite treatment and eggs were plated on NGA plates spotted with *E*. *coli* strain OP50-1 and grown to adulthood at 20°C. Starting at 48 hours after synchronization, worms were scored for development to adulthood by microscopy every 8–16 hours until the entire population had reached adulthood. Three plates of approximately 100 worms each were scored for each genotype.

### Stress assays

For heat stress assays, N2 and outcrossed *rpn-10(ok1865)* worms were synchronized via hypochlorite treatment and grown to the L4 stage on *E*. *coli* OP50-1-spotted NGA plates at 20°C. Forty L4 individuals of each strain were then transferred to fresh plates in duplicate and incubated at 35°C. Scoring for survival was performed every hour beginning six hours after initiation of heat stress, with dead worms being identified by lack of responsiveness to gentle prodding with a pick.

Oxidative stress assays using *tert-*butyl hydroperoxide (tBHP) were performed as described with 40 L4 animals being transferred to *E*. *coli* OP50-1-spotted NGA plates containing 7mM tBHP and scored three times per day using the parameters detailed above until all animals died or were censored [122]. A minimum of two trials with comparable results were performed for each assay.

Oxidative stress assays using 5-Hydroxy-1,4-naphthoquinone (juglone) were performed using WT and *rpn-10* worms which were treated with control and *skn-1* RNAi from egg hatching for 48 hours. The worms were then washed from plates and suspended in M9 buffer. Juglone was added to a final concentration of 100 μM from a 100X stock made fresh in 100% ethanol, and the worms were then exposed to juglone for one hour with nutation. The worms were then washed twice with S-basal and returned to NGA. They were scored for survival 48 hours later.

### Polyglutamine-repeat protein aggregation assays

The aggregation of the muscle-expressed Q35::YFP fusion protein was assessed by adding eggs isolated from *rmIs132[unc-54p*::*Q35*::*YFP]* and *rpn-10(ok1865); rmIs132[unc-54p*::*Q35*::*YFP]* animals via hypochlorite treatment to NGA plates, and then incubating the plates at 23°C for 72 hours [112]. After this time essentially all of the animals were gravid adults. The number of individual Q35::YFP aggregates were scored using a fluorescent stereomicroscope as previously described, and digital images were captured at 6X magnification using a Nikon Eclipse inverted compound microscope equipped with epifluorescence illumination and a Nikon Endow GFP filter cube [26]. The aggregation of the intestine-expressed Q44::YFP fusion protein was assessed by adding eggs isolated from *drIs20[vha-6p*::*Q44*::*YFP + rol-6(su1006)]* and *rpn-10(ok1865); drIs20[vha-6p*::*Q44*::*YFP + rol-6(su1006)]* animals via hypochlorite treatment to NGA plates, and then incubating the plates at 23°C for 72 hours [48]. At this point ~100 worms were transferred to either NGA plates containing 50 μM FUDR or RNAi plates containing 50 μM FUDR and spotted with the indicated RNAi clone, and the plates were returned to 23°C for 4 days before scoring. The *drIs20* worms contained large numbers of aggregates at this point, so the percentage of animals with any aggregates present in the intestine was measured by scoring with a fluorescent microscope. For S9 Fig, we also counted individual aggregates in the *drIs20* worms, and to enhance the aggregate numbers in the *rpn-10* mutant animals, we incubated the plates at 23°C for 5 days instead of 4 days. The aggregate numbers were scored using a fluorescent microscope after briefly incubating the plates on ice to reduce worm activity.

### Temperature-shift paralysis assays

A previously described protocol for assessing the phenotypic effects of shifts from permissive to non-permissive temperature on the function of the meta-stable UNC-54 protein in the *unc-54(e1157)* mutant was adapted for the analysis of larval animals [49]. Briefly, synchronized L1 larval populations of *unc-54(e1157)*, and *rpn-10*, *unc-54* animals were spotted on NGA plates and grown at 16°C (permissive temperature) for 24 hours. At this time, a total of approximately 300 worms per genotype were transferred to three separate fresh NGA plates, and shifted to 25°C (non-permissive temperature) for 20 hours. The plates were then allowed to equilibrate at room temperature for 20 minutes before being scoring for paralysis by prodding with a platinum worm-pick. Worms that failed to respond to touch were scored as paralyzed.

### Transcription factor RNAi library screens

Two transcription factor RNAi libraries were independently screened to identify transcription factors that permitted survival of the *rpn-10(ok1865)* mutant. These libraries were (1) a subset library sold by Source Bioscience which was created from Ahringer RNAi library clones, and (2) a library created from clones in the Ahringer and Vidal RNAi libraries (S6 Table). For each screen, individual wells of 24-well plates containing NGA with 50μg/ml carbenicillin and 0.2% β-lactose were spotted with 20μL of overnight culture for a clone in the transcription factor library and allowed to dry at room temperature. Each well was then seeded with 70–100 *rpn-10(ok1865); zcIs1[aip-1*::*GFP]* L1 larvae, which had hatched from eggs that were isolated by hypochlorite treatment and then placed in S-basal to arrest the progeny at the L1 larval stage. Each plate was then incubated at 20°C and visually screened for phenotypic effects after three days. Clones that caused developmental arrest or sickness were then re-screened, and clones that were again found to produce these phenotypes in the second round were used to treat larger populations of *zcIs1[aip-1*::*GFP]* animals both with and without the *rpn-10(ok1865)* allele in order to find genes required for the development and survival of mutant but not wild-type animals. In this manner clones determined to be hits from the initial screen were sequentially narrowed down to those that consistently impaired normal development only in the *rpn-10(ok1865)* background.

### Measurement of gene expression by Nanostring

Code sets that recognize the indicated genes were synthesized by Nanostring Technologies (Seattle, WA) and used with the Nanostring nCounter system to measure the levels of each transcript in 100 ng aliquots of total RNA. The resulting nCounter data were analyzed using the Nanostring nSolver data analysis software with normalization to the geometric mean of the level of the *cdc-42*, *pmp-3*, and *Y45F10D*.*4* transcripts in each sample [123]. The normalized expression data were then exported to MS Excel for further analysis.

RNA for Nanostring studies was isolated from wild-type (N2) or *rpn-10(ok1865)* animals grown from egg hatching on control, *skn-1*, or 1:10 diluted *elt-2* RNAi for 48 hours. This time point was selected because no visible differences in worm morphology were observed in any of the treatment groups, so any changes in gene expression likely occurred before the animals became ill due to RNAi treatment. After washing the animals from the plates in S-basal, the worm pellet was then suspended in QIAzol lysis reagent and frozen at -80°C. Total RNA was isolated using the Qiagen miRNeasy kit. The yield and quality of each RNA sample was evaluated using a Nanodrop spectrophotometer and also by running an aliquot on an Agilent Bioanalyzer. For each genotype-RNAi treatment pair, six biological replicates were performed.

### Whole transcriptome RNA sequencing

Three independent populations of N2 control and *rpn-10(ok1865)* mutant worms were synchronized via the use of hypochlorite treatment and grown on *E*. *coli* OP50-1 spotted NGA plates at 20°C for 3 days. The worms were then washed from the plates and washed twice with milliQ-purified water. The worm pellet was then suspended in QIAzol lysis reagent and frozen at -80°C. Total RNA was isolated using the Qiagen miRNeasy mini kit, and the RNA yield was measured by spectrophotometry. Total RNA was sent to Expression Analysis (Durham, NC) for analysis including Agilent Bioanalyzer electrophoresis to ensure RNA quality followed by library preparation using the Illumina TruSeq RNA sample prep kit. The resulting library was subjected to high-throughput 50 nucleotide paired end sequencing using an Illumina sequencer at a depth of 17 million reads per sample.

The resulting sequence data were analyzed as previously described [119]. Briefly, the sequence reads were clipped using internally developed software by Expression Analysis and matched to the *C*. *elegans* genome using RSEM [124]. The resulting transcript counts were then normalized using the upper quartile normalization approach [125]. Differentially expressed genes were then identified through the use of serial *t-*testing coupled with Benjamini-Hochberg correction and genes with an adjusted p-value score less than 0.05 were considered to be differentially expressed. This led to the identification of 171 genes as being differentially expressed (111 up-regulated and 60 down-regulated) between *rpn-10(ok1865)* and wild-type N2 (S2 and S3 Tables). Over-represented gene classes were identified in the up-regulated and down-regulated genes through the use of DAVID [126].

### Lysosome staining

Lysosomes were stained via two complementary approaches. The first approach utilized the Lysotracker Red stain (Life Technologies #L7528) and Lysosensor Green stain (Life Technologies #L7535), which concentrate in the low pH environment of the lysosome, while the second utilized the Magic Red cathepsin B and cathepsin L substrates (ImmunoChemistry Technologies #938 and #942) [89]. These cathepsin B and L substrates are cell-permeable cresyl violet-conjugated peptides containing either the Arg-Arg or Phe-Arg sequence cleaved by the respective cathepsin in the lysosome, and this cleavage event relieves an intramolecular quenching of the cresyl violet fluorophore and produces red fluorescence.

Lysotracker Red and Lysosensor Green staining were both performed by spotting NGA plates with an aliquot of dye from a 1mM working stock diluted in S-basal to produce a final concentration of 2 μM [127]. The spotted plates were allowed to dry for one hour at room temperature before L4 larval animals were added. The worms were stained overnight at 20°C, and then transferred to unspotted NGA plates for one hour to clear residual dye from the intestinal lumen. The animals were then mounted on slides and imaged using a Nikon Eclipse Ti inverted microscope using a Y-2E/C filter cube. Images were captured at 20X magnification using a CoolSNAP HQ2 (Photometrics) CCD camera and Nikon Elements software. Fluorescence intensity was measured using ImageJ [120]. We did not stain the control and *elt-2* RNAi treated animals with Lysotracker Red because preliminary experiments demonstrated greater penetration of Lysotracker into the *elt-2* RNAi treated animals. Particularly, we observed the staining of tissues, like the hypodermis, that are not seen in animals stained on control RNAi or NGA, which suggested that the absorption or distribution of the dye is not similar between the RNAi treatments thus precluding reliable comparisons.

Magic Red staining was performed by spotting NGA, control RNAi, or *elt-2* RNAi plates with an aliquot of dye from a 260X stock, prepared by dissolving the powdered dye in DMSO following the manufacturer’s instructions, to give a final 1X concentration. To facilitate spreading of the dye on the plate, the aliquot was mixed with water to produce a final volume of 20 μL prior to pipetting onto the plate. To conserve dye, we performed these experiments in 12 well plates containing 3 mL of agar per well. The plates were allowed to dry for one hour at room temperature before L4 larval animals were added. The animals were incubated at 20°C overnight, and then cleared of residual dye and imaged as described above.

## Supporting Information

S1 Table Lifetables for lifespan experiments. (XLSX)

S2 Table Genes identified as differentially expressed via whole transcriptome analysis (RNA-seq) studies comparing wild-type and *rpn-10(ok1865)* mutant animals. (XLSX)

S3 Table Over-represented gene classes identified among the up-regulated genes via the use of the DAVID program. (XLSX)

S4 Table Effects of the *rpn-10(ok1865)* mutant on genes involved in oxidative stress responses, heat shock responses, proteasome function, and autophagy. (XLSX)

S5 Table Statistical values for oxidative stress and proteasome subunit gene expression level changes in the rpn-10 mutant for Fig 5D and 5E. (XLSX)

S6 Table RNAi clones present in the combined transcription factor library made by utilizing clones from the Ahringer and Vidal RNAi libraries. (XLS)

S1 Fig Expression pattern of RPN-10::GFP. Panels A-H display additional images of wild-type animals expressing an RPN-10::GFP fusion protein which shows expression in multiple tissues including the excretory cell, somatic gonad, hypodermis, intestine, body wall muscle, pharynx, and vulva.
(TIF)

S2 Fig Quantification of mCherry fluorescence from Fig 1E. (TIF)

S3 Fig The *rpn-10* mutation causes graded proteasome dysfunction. The *rpn-10* mutation does not cause accumulation of UbV::GFP until day 1 of adulthood, while RNAi for several other proteasome subunits, *pas-6*, *pbs-6*, and *pbs-7* cause high UbV::GFP accumulation during larval development.
(TIF)

S4 Fig Expression pattern of *aip-1p*::*GFP*. Panels A-H display additional images of *rpn-10* mutant animals expressing an *aip-1p*::*GFP* transgene which shows expression in multiple tissues including the intestine, pharynx, body wall muscle, excretory cell, somatic gonad, and hypodermis.
(TIF)

S5 Fig The *rpn-10* mutant shows a decrease in Q35::YFP aggregation. Adult mutant worms consistently show far less accumulation of Q35::YFP aggregates than wild-type animals.
(TIF)

S6 Fig The *rpn-10* mutant shows a decrease in Q44::YFP aggregation. Adult mutant worms consistently show little to no aggregation of Q44::YFP compared to wild-type animals expressing the same transgene.
(TIF)

S7 Fig The *rpn-10* mutants treated with *skn-1* and *elt-2* RNAi appear normal on day 2 of development and then develop the small and sickly phenotypes during late larval development. The *rpn-10* mutant RNAi-treated worms develop normally compared to the *rpn-10* mutant treated with control RNAi when visualized on the second day after synchronization, but then are small and sickly when examined on the third day when the control RNAi treated animals are adults.
(TIF)

S8 Fig The *rpn-10* mutant shows increased expression of *lgg-1* and *bec-1* via the use of Nanostring analysis. In Panel A, * represents p = 0.043 by *t*-test, and in panel B, *** represents p = 0.002 by *t*-test. Of note, the changes in *lgg-1* and *bec-1* expression are independent of *skn-1* and *elt-2*.
(TIF)

S9 Fig RNAi directed against *atg-13*, *atg-16*.*2*, and *prmt-1* have a greater effect on Q44::YFP aggregation in the *rpn-10* mutant than in wild-type animals expressing the same transgene. (A) The treatment of wild-type worms expressing the Q44::YFP transgene results in only modest increases in the percentage of animals with aggregates compared to the control RNAi treated animals. (B) Similarly, counting the number of aggregates per worm reveals a relatively greater increase in the number of aggregates in the *rpn-10* mutant animals compared to the wild-type animals. Specifically, the presence of the *rpn-10* mutation reduces the number of aggregates 11.5 fold in the control RNAi treated worms compared to only 3.7 fold in the *atg-13* RNAi treatment, 3.9 fold in the *atg-16*.*2* RNAi treatment, and 6.2 fold in the *prmt-1* RNAi treatment (n = 50 worms for all genotypes and treatments except n = 41 for *rpn-10 –*control RNAi, n = 28 for *rpn-10 –atg-13* RNAi, and n = 42 for WT–*atg-16*.*2* RNAi). (C) A cumulative plot of aggregate number which plots the animal number, ranked from lowest aggregate count to highest, on the y-axis versus the number of aggregates on the x-axis reveals the shift towards having fewer animals with no aggregates and a subset of animals with a collapse in proteostasis as indicated by very high aggregate counts in the *atg-13*, *atg-16*.*2*, and *prmt-1* RNAi treated *rpn-10* mutant animals compared to the control RNAi treatment. In contrast the curves for the wild-type animals treated with the same RNAi clones only results in slight shifts of curves with an overall similar shape.
(TIF)

S10 Fig The Lysosensor Green and Magic Red stain for cathepsin B activity show extensive co-localization. Wild-type N2 worms were stained with both dyes and then mounted for fluorescent microscopy.
(TIF)

S11 Fig The role of *skn-1* in the enhanced oxidative stress resistance of the *rpn-10* mutant. The oxidative stress resistance of the *rpn-10* mutant to the pro-oxidant juglone is significantly decreased when treated with *skn-1* RNAi (n = 100 for *rpn-10* treated with control or *skn-1* RNAi, p<0.0001 by Fisher’s exact test).
(TIF)
