# Supplementary figures and images for: Graded Proteasome Dysfunction in Caenorhabditis elegans Activates an Adaptive Response Involving the Conserved SKN-1 and ELT-2 Transcription Factors and the Autophagy-Lysosome Pathway
Source: PLoS Genet. 2016 Feb 1;12(2):e1005823. doi: 10.1371/journal.pgen.1005823 (PMC4734690; doi:10.1371/journal.pgen.1005823)

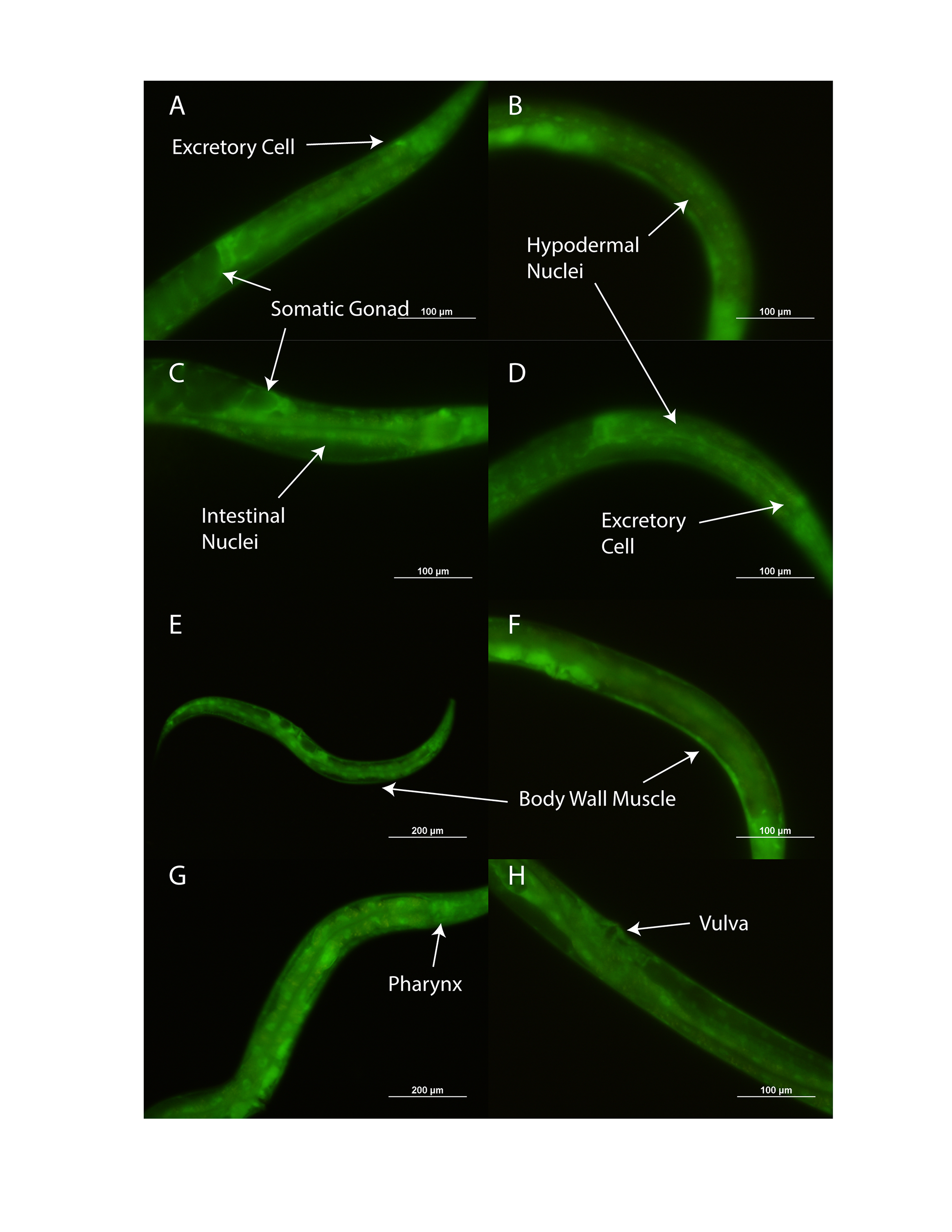

Supplement: S1 Fig — Panels A-H display additional images of wild-type animals expressing an RPN-10::GFP fusion protein which shows expression in multiple tissues including the excretory cell, somatic gonad, hypodermis, intestine, body wall muscle, pharynx, and vulva. (TIF) [file pgen.1005823.s007.tif]

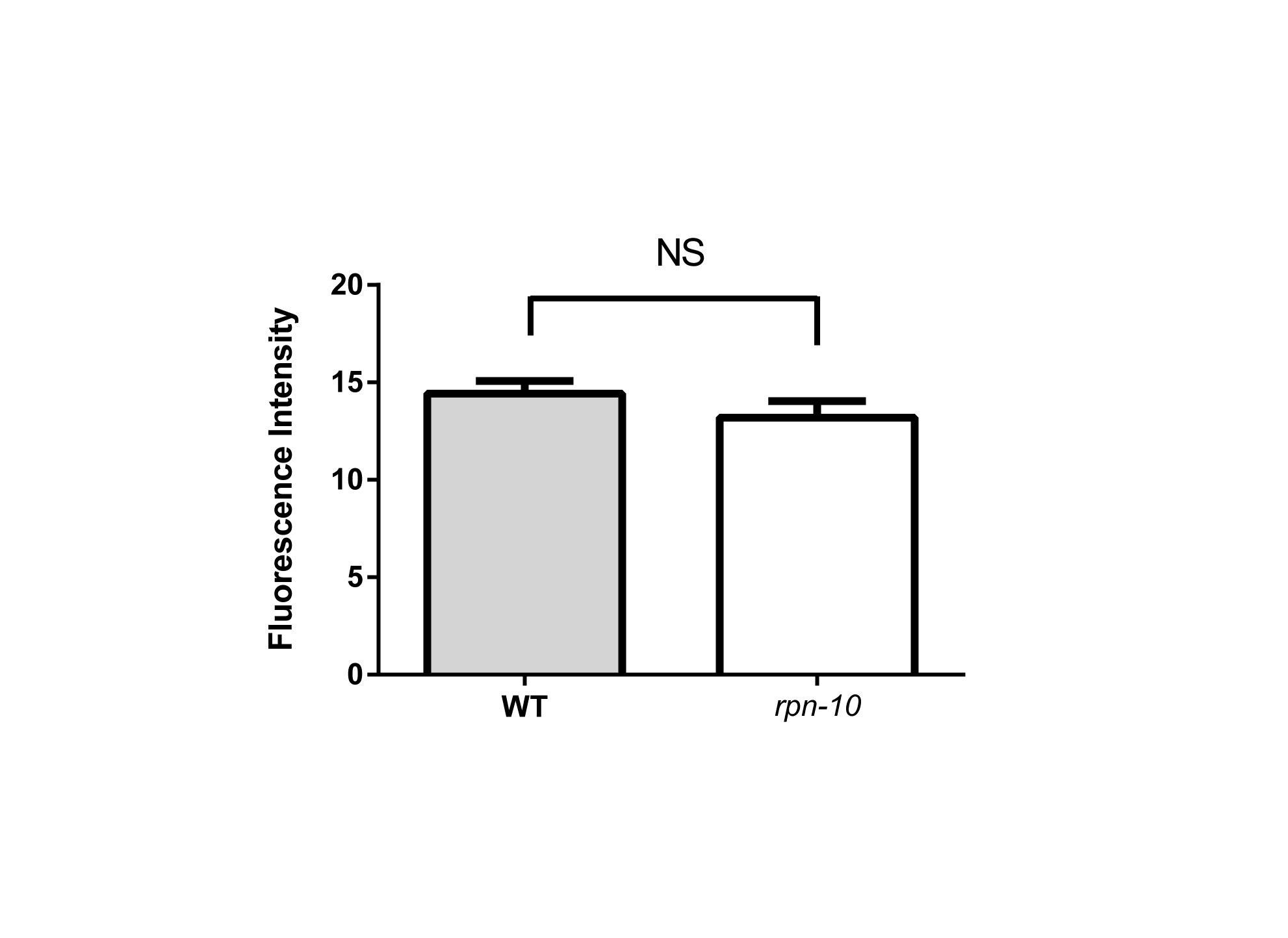

Supplement: S2 Fig — (TIF) [file pgen.1005823.s008.tif]

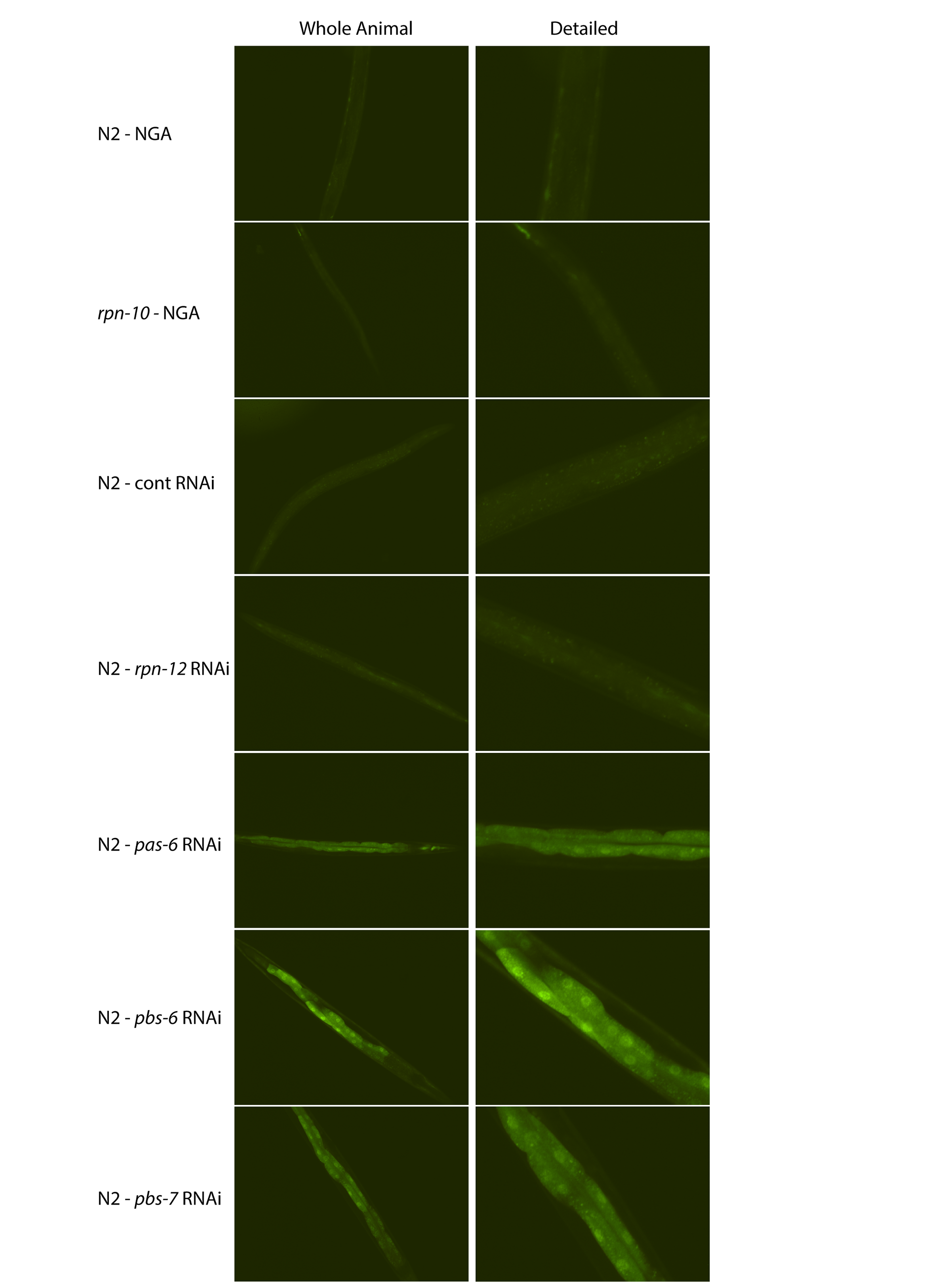

Supplement: S3 Fig — The rpn-10 mutation does not cause accumulation of UbV::GFP until day 1 of adulthood, while RNAi for several other proteasome subunits, pas-6, pbs-6, and pbs-7 cause high UbV::GFP accumulation during larval development. (TIF) [file pgen.1005823.s009.tif]

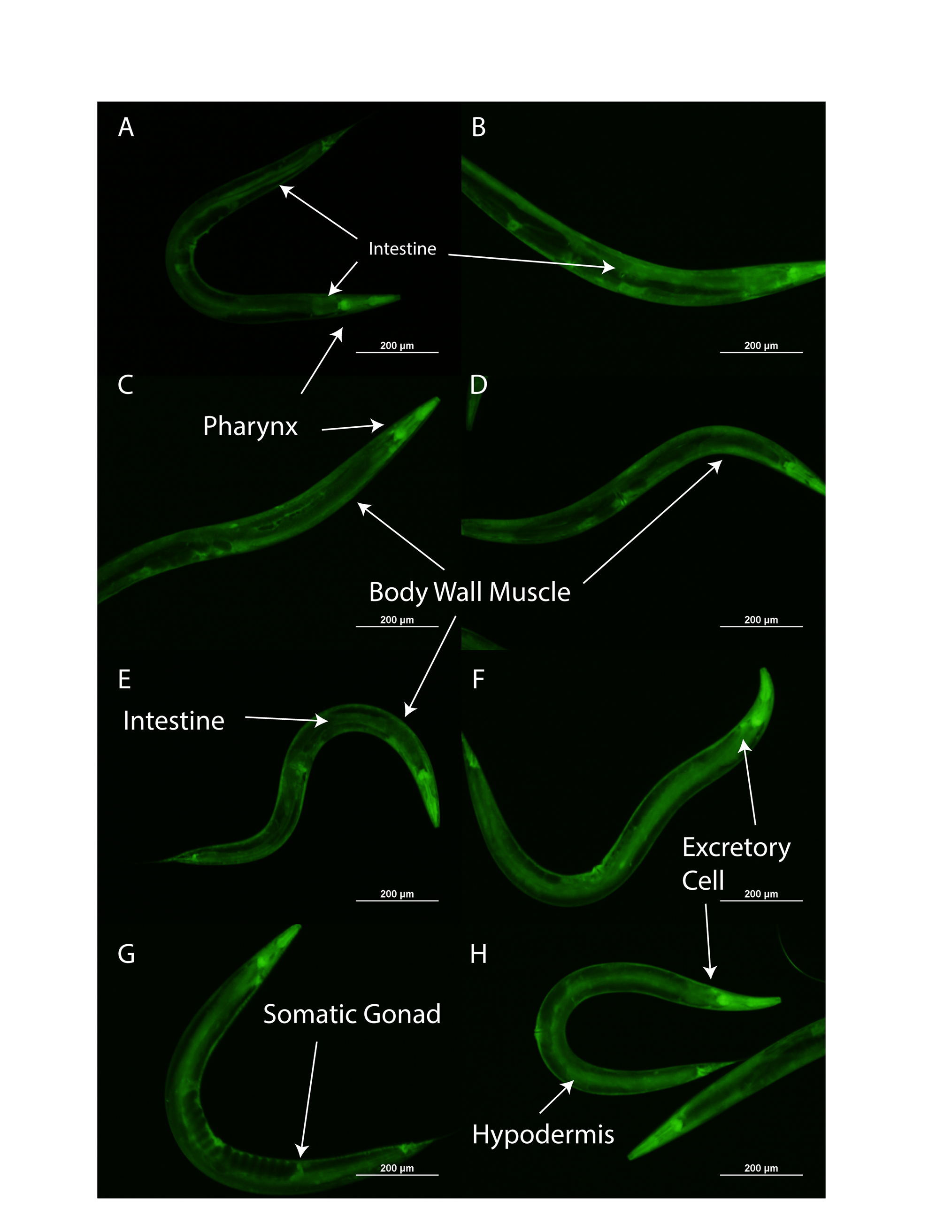

Supplement: S4 Fig — Panels A-H display additional images of rpn-10 mutant animals expressing an aip-1p::GFP transgene which shows expression in multiple tissues including the intestine, pharynx, body wall muscle, excretory cell, somatic gonad, and hypodermis. (TIF) [file pgen.1005823.s010.tif]

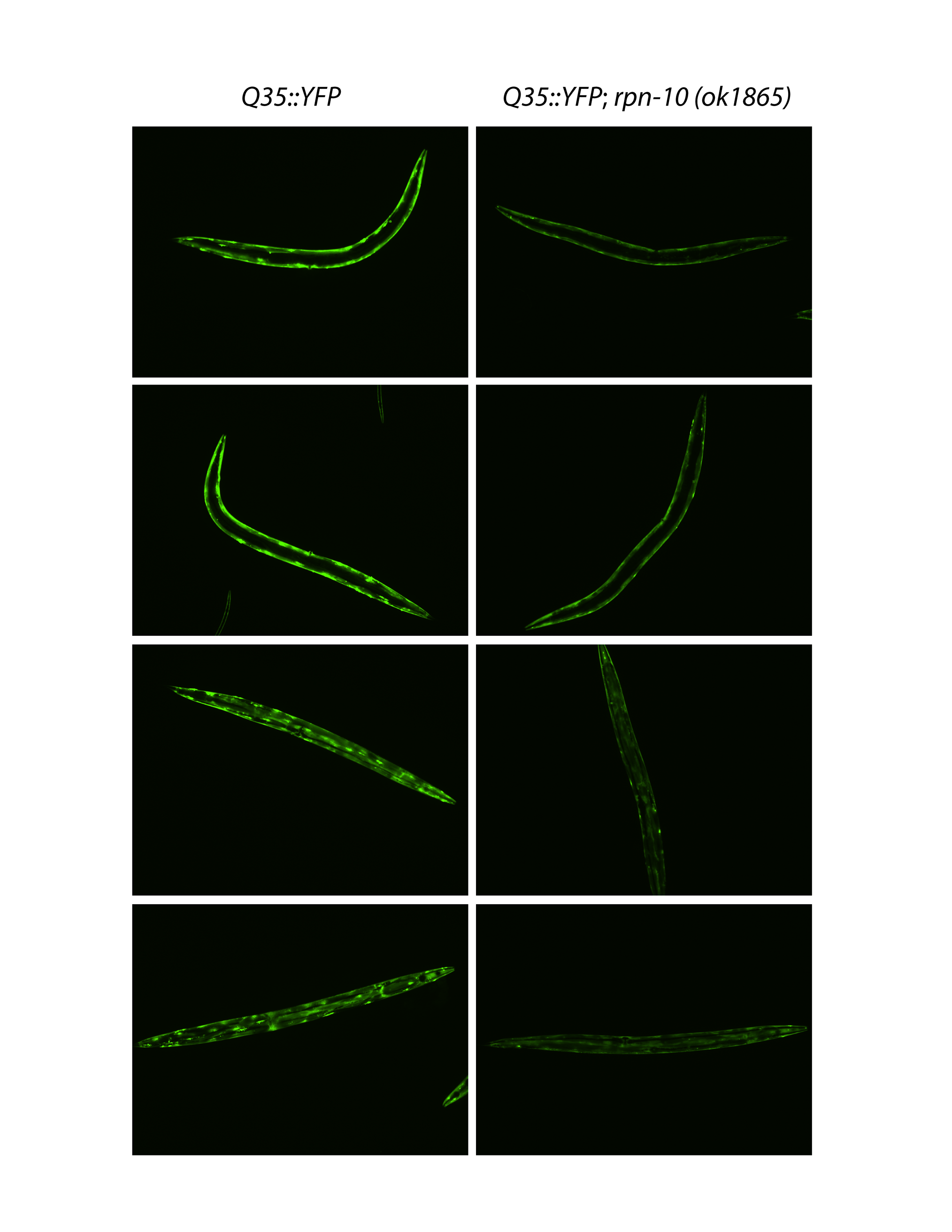

Supplement: S5 Fig — Adult mutant worms consistently show far less accumulation of Q35::YFP aggregates than wild-type animals. (TIF) [file pgen.1005823.s011.tif]

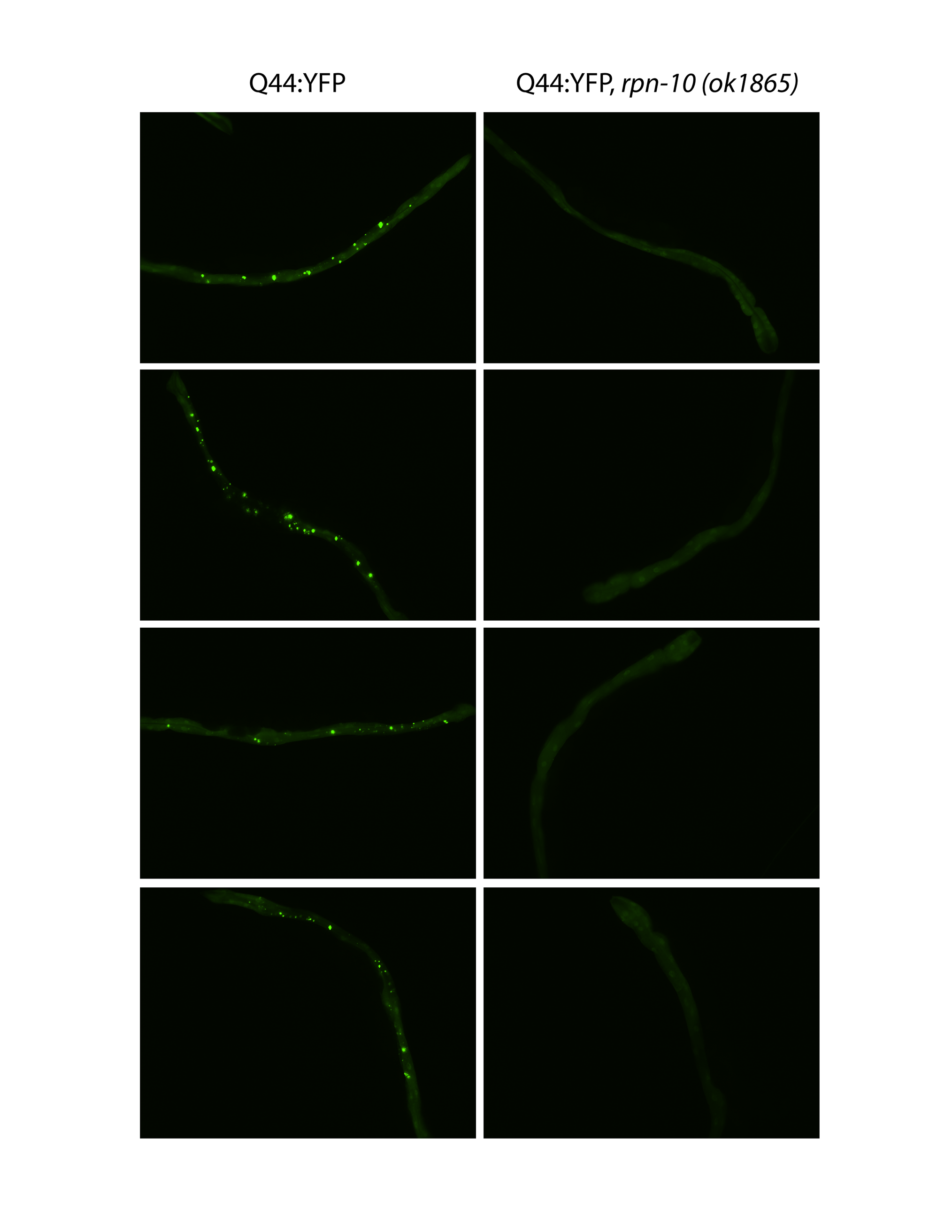

Supplement: S6 Fig — Adult mutant worms consistently show little to no aggregation of Q44::YFP compared to wild-type animals expressing the same transgene. (TIF) [file pgen.1005823.s012.tif]

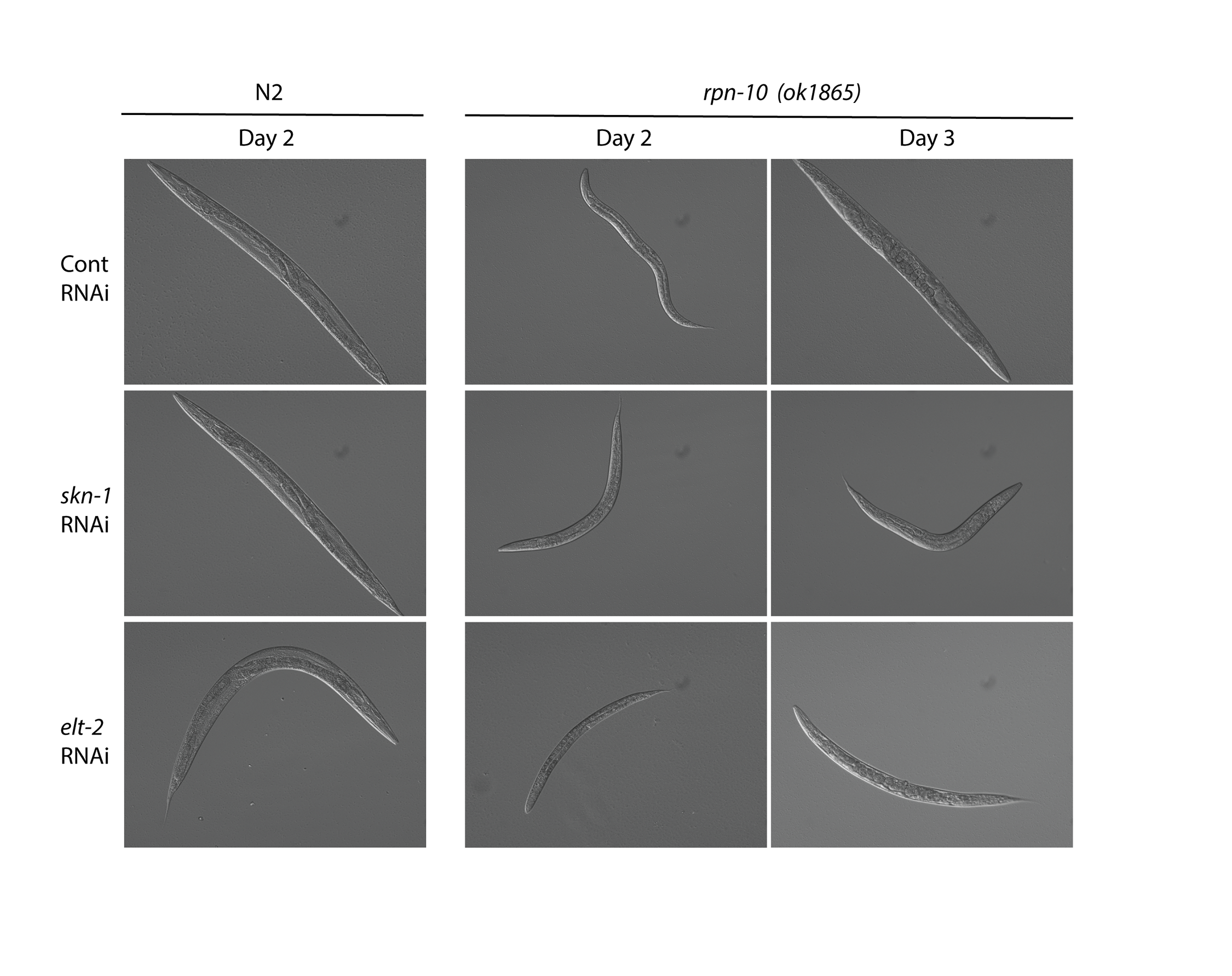

Supplement: S7 Fig — The rpn-10 mutant RNAi-treated worms develop normally compared to the rpn-10 mutant treated with control RNAi when visualized on the second day after synchronization, but then are small and sickly when examined on the third day when the control RNAi treated animals are adults. (TIF) [file pgen.1005823.s013.tif]

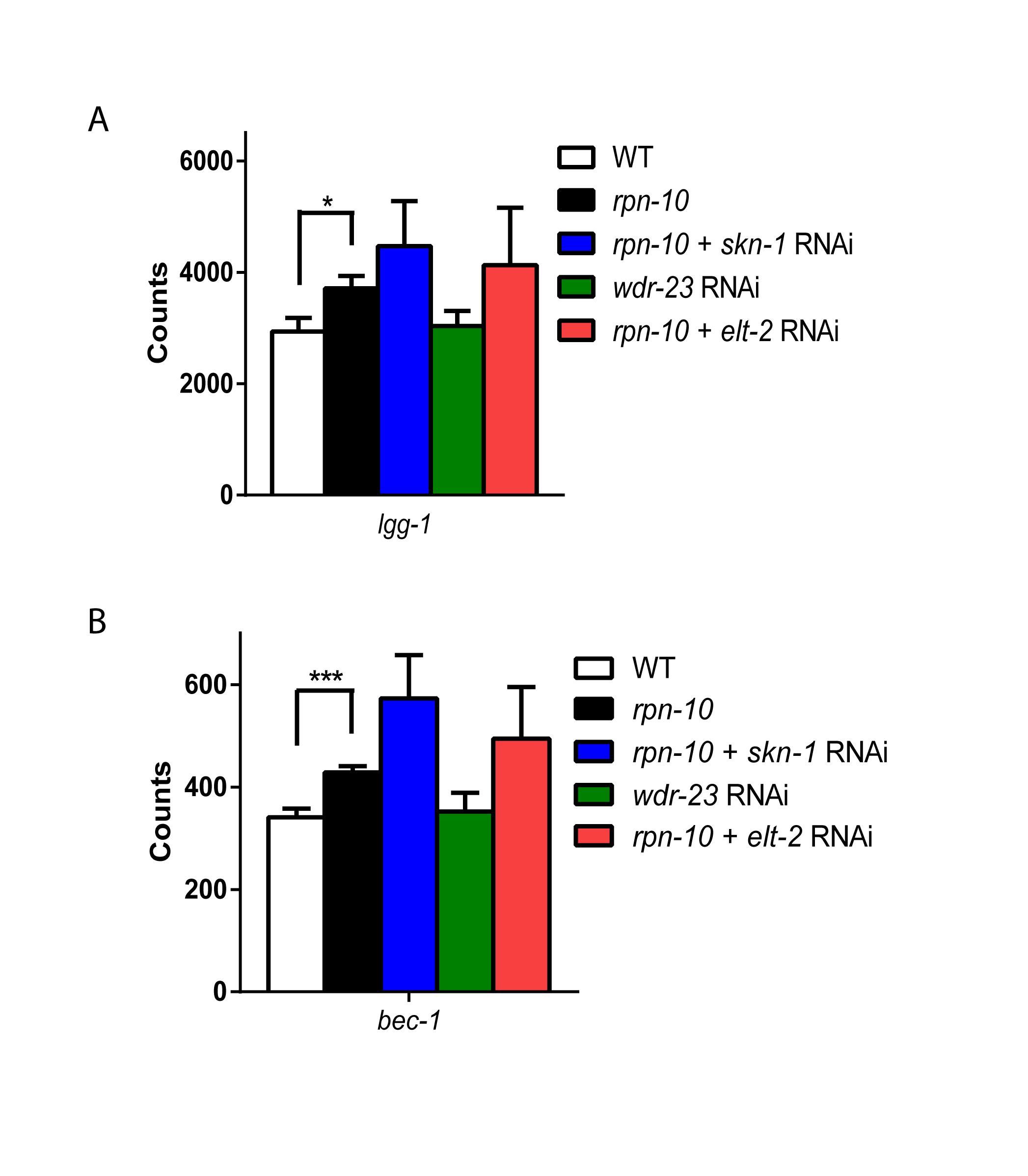

Supplement: S8 Fig — In Panel A, * represents p = 0.043 by t-test, and in panel B, *** represents p = 0.002 by t-test. Of note, the changes in lgg-1 and bec-1 expression are independent of skn-1 and elt-2. (TIF) [file pgen.1005823.s014.tif]

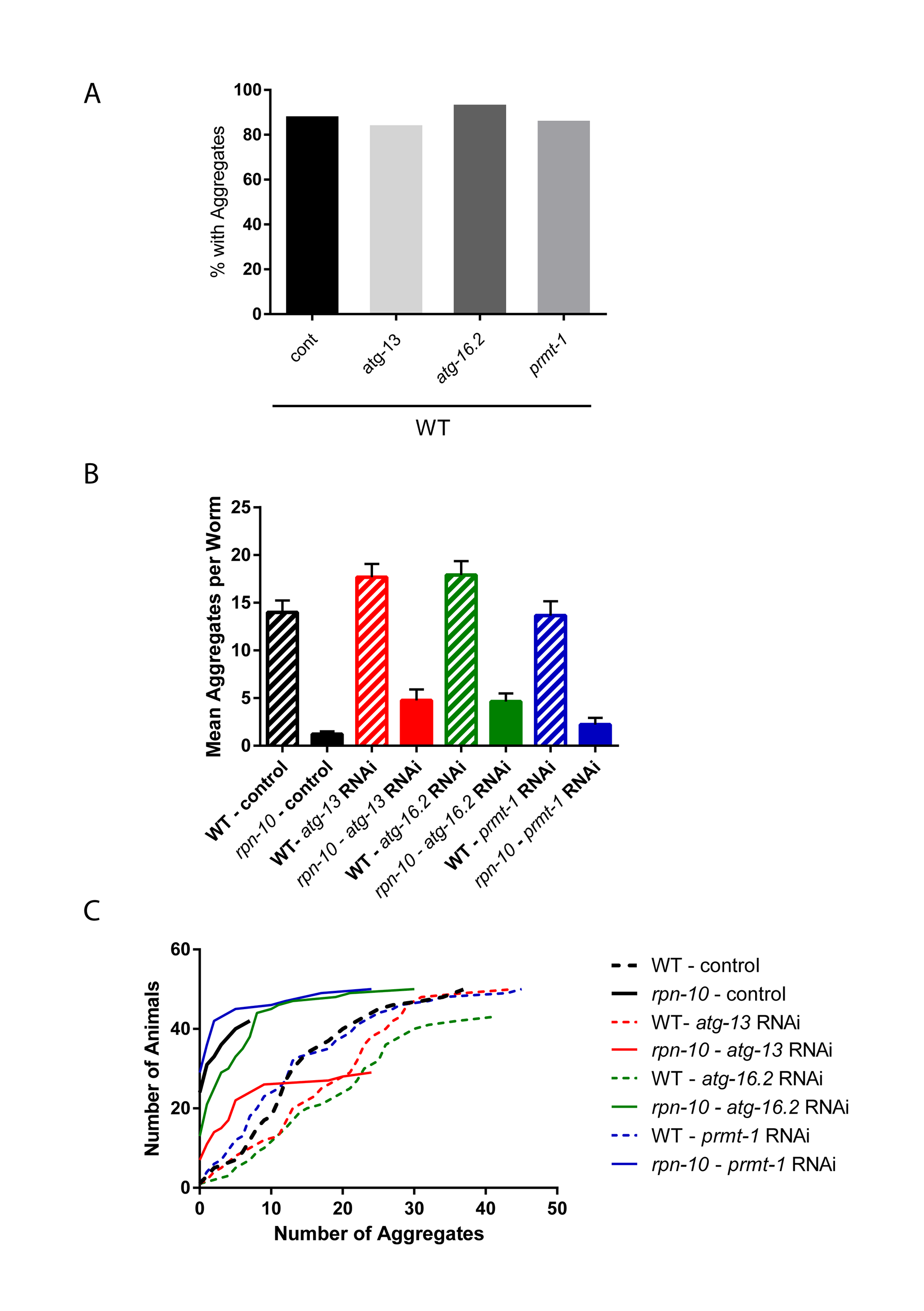

Supplement: S9 Fig — (A) The treatment of wild-type worms expressing the Q44::YFP transgene results in only modest increases in the percentage of animals with aggregates compared to the control RNAi treated animals. (B) Similarly, counting the number of aggregates per worm reveals a relatively greater increase in the number of aggregates in the rpn-10 mutant animals compared to the wild-type animals. Specifically, the presence of the rpn-10 mutation reduces the number of aggregates 11.5 fold in the control RNAi treated worms compared to only 3.7 fold in the atg-13 RNAi treatment, 3.9 fold in the atg-16.2 RNAi treatment, and 6.2 fold in the prmt-1 RNAi treatment (n = 50 worms for all genotypes and treatments except n = 41 for rpn-10 –control RNAi, n = 28 for rpn-10 –atg-13 RNAi, and n = 42 for WT–atg-16.2 RNAi). (C) A cumulative plot of aggregate number which plots the animal number, ranked from lowest aggregate count to highest, on the y-axis versus the number of aggregates on the x-axis reveals the shift towards having fewer animals with no aggregates and a subset of animals with a collapse in proteostasis as indicated by very high aggregate counts in the atg-13, atg-16.2, and prmt-1 RNAi treated rpn-10 mutant animals compared to the control RNAi treatment. In contrast the curves for the wild-type animals treated with the same RNAi clones only results in slight shifts of curves with an overall similar shape. (TIF) [file pgen.1005823.s015.tif]

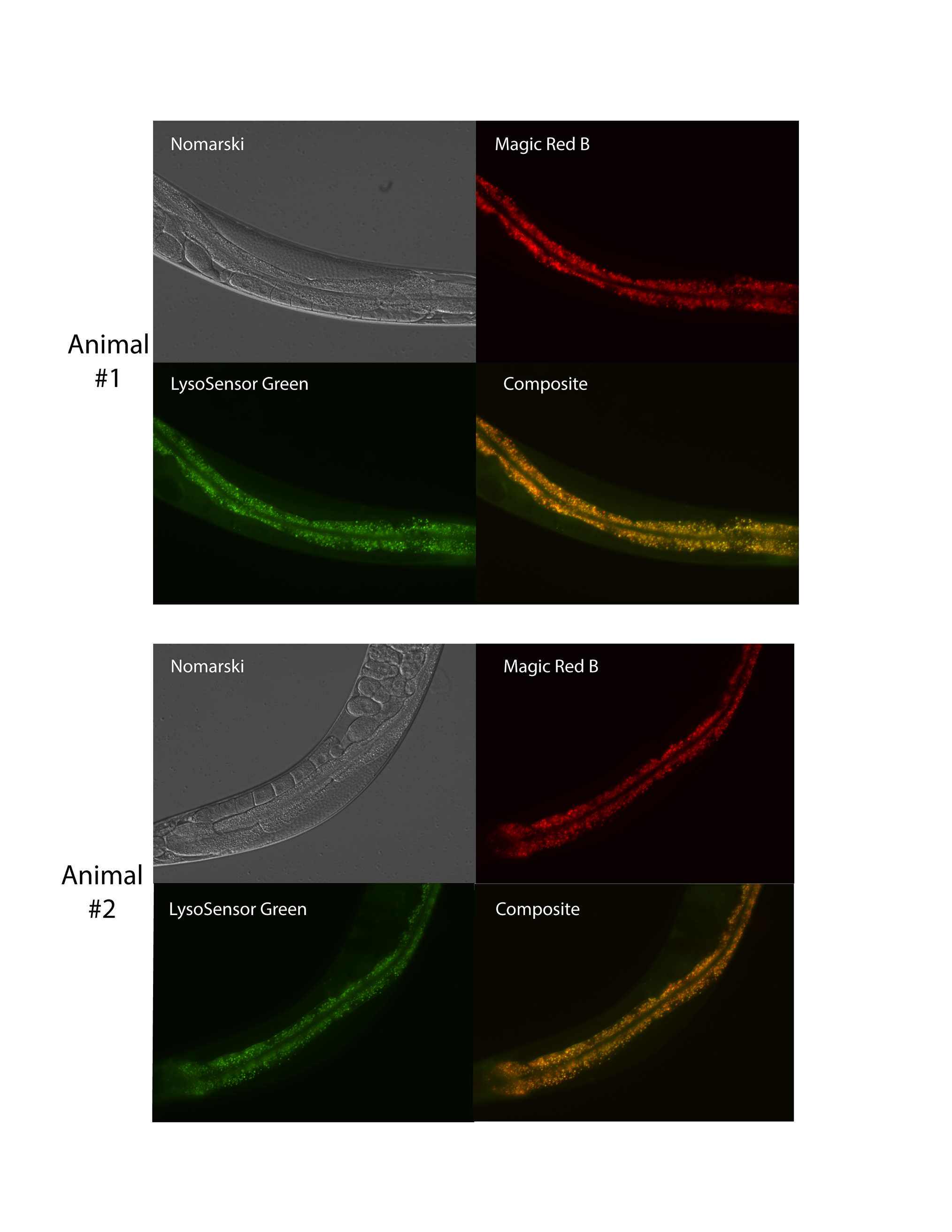

Supplement: S10 Fig — Wild-type N2 worms were stained with both dyes and then mounted for fluorescent microscopy. (TIF) [file pgen.1005823.s016.tif]

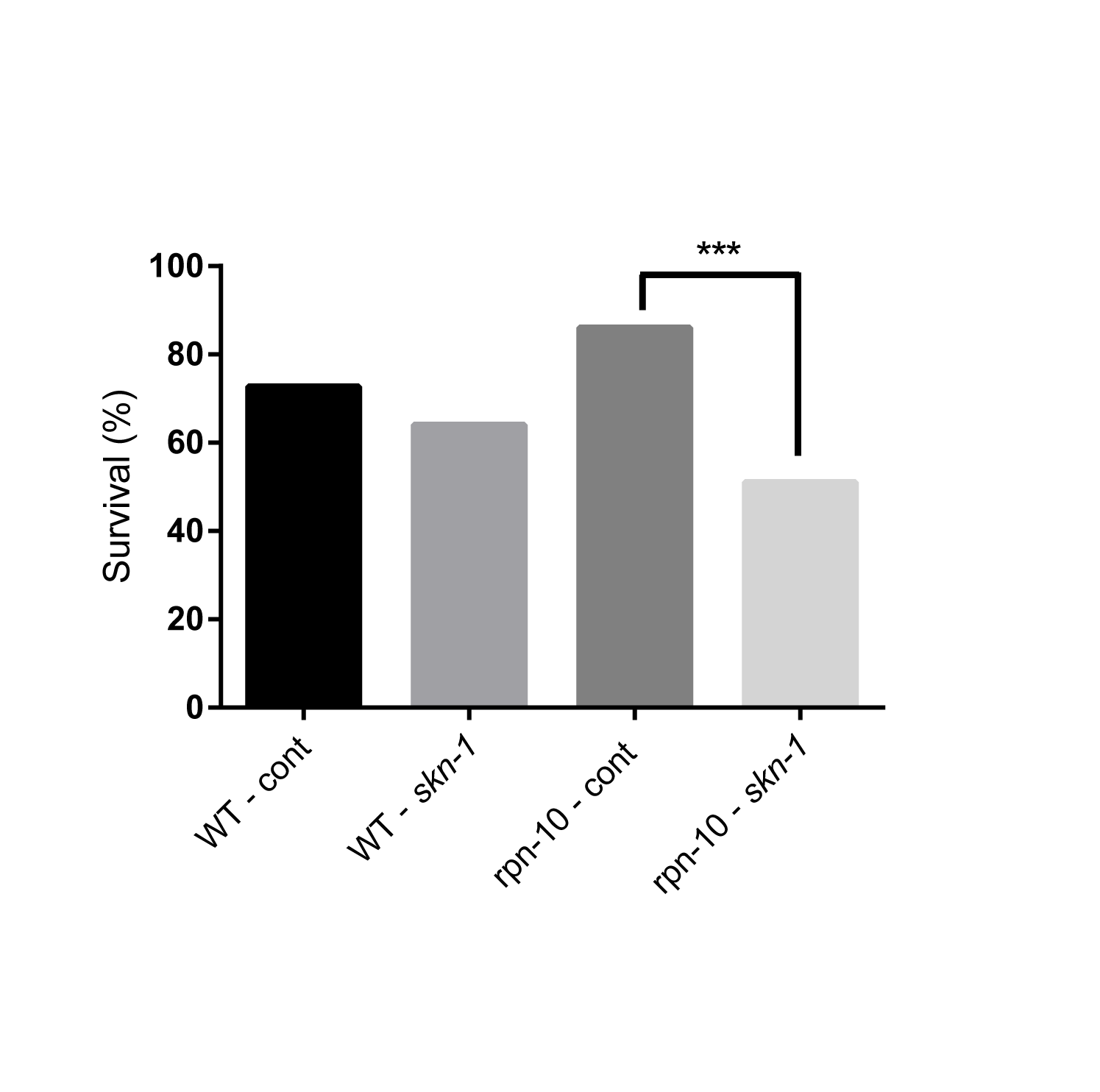

Supplement: S11 Fig — The oxidative stress resistance of the rpn-10 mutant to the pro-oxidant juglone is significantly decreased when treated with skn-1 RNAi (n = 100 for rpn-10 treated with control or skn-1 RNAi, p<0.0001 by Fisher’s exact test). (TIF) [file pgen.1005823.s017.tif]
